# Using human-centered design to advance health literacy in local health department programming: a case study

**DOI:** 10.1186/s12889-025-22491-z

**Published:** 2025-03-31

**Authors:** Adriane Ackerman, Brittany Nigon, Alexis Wait, Elham Ali, Ada M. Wilkinson-Lee, Alexia Cohen, Meredith Jones, Imelda G. Cortez, Katrina Kelly, Robert Fabricant, Jenitza Serrano-Feliciano, Jennifer Stanowski, Theresa Cullen

**Affiliations:** 1 Agile Accomplice LLC, 2714 N Los Altos Ave, Tucson, AZ 85705 USA; 2 Pima County Health Department, 3950 S Country Club Rd, Suite 100, Tucson, AZ 85714 USA; 3 Johns Hopkins Bloomberg School of Public Health, 615 N Wolfe St, Baltimore, MD 21205 USA; 4 Department of Mexican American Studies, The University of Arizona, P.O. Box 210023, Tucson, AZ 85721-0023 USA; 5 Dalberg Design, 155 W 23rd St, New York, NY 10011 USA; 6 MHC Healthcare, 13395 N Marana Main St, Marana, AZ 85653 USA; 7 The Behavioral Insights Team, 1 Dock 72 Way, 7th Floor, Brooklyn, NY 11205 USA; 8 Literacy Connects, 200 E Yavapai Rd, Tucson, AZ 85705 USA

**Keywords:** Human-centered design, Health literacy, Public health, Participatory design, Inclusion, Equity, Community health workers

## Abstract

**Background:**

Human-centered design (HCD) and behavioral science are structured, evidence-based methodologies used to develop and evaluate community-driven interventions. While HCD focuses on deeply understanding user needs and co-designing solutions, behavioral science applies empirically tested principles to drive behavior change. Together, these methodologies enable the development of interventions that are both user-centered and behaviorally informed. The Pima County Health Department and project partners leveraged these collaborative methodologies to assemble a Community of Practice to improve health literacy and adherence to COVID-19 public health practices among Hispanic/Latine individuals of childbearing age and ability in Pima County.

**Methods:**

Human-centered design processes identified and evaluated barriers facing the target population. On the basis of these findings, two pilot interventions were implemented between July 2023 and November 2023: one in a clinical setting with 92 participants and another in a community setting with 207 participants. A mixed-methods approach was used to evaluate the impact of these pilots. Quantitatively, a pre-post evaluation and survey design estimated the effect of an intervention by comparing outcomes before and after implementation using paired t-test and chi-square tests. Qualitatively, structured post intervention interviews were conducted with participants who were randomly selected based upon their initial consent and willingness to participate.

**Results:**

Participants in the clinical and community pilots perceived fewer barriers to health-seeking behaviors after the intervention. Both pilots increased participants’ confidence in health-seeking behaviors (*p* < 0.01). Only the clinical pilot resulted in an increase in health literacy. In the clinical pilot, the number of unvaccinated participants decreased, and the number of participants who reported needing a booster increased. The community pilot did not find a statistically significant difference in COVID-19 vaccine uptake.

**Conclusions:**

Integrating human-centered design and behavioral science into public health interventions can improve health literacy and confidence in health-seeking behaviors among historically and contemporarily excluded populations. Local health departments can use these methods to develop multicomponent interventions that foster mutual co-invention with communities and improve population health outcomes. Future research should focus on long-term impacts and explore broader applications of these approaches in different contexts.

**Trial registration:**

This project received University of Arizona IRB review and approval. This study was not considered a randomized controlled trial and did not require registration.

**Supplementary Information:**

The online version contains supplementary material available at 10.1186/s12889-025-22491-z.

## Background

In 2020, the US Office of Disease Prevention and Health Promotion amended the definition of health literacy in the Healthy People 2030 framework to include both personal and organizational health literacy. Traditional definitions of health literacy have focused on an individual’s ability to seek, comprehend, and utilize health-related information to make informed decisions. However, this perspective often overlooks sociocultural and systemic barriers that affect a person’s ability to improve their own health literacy. In response, the expanded definition acknowledges that organizations also bear responsibility for fostering health literacy and ensuring that individuals can easily access and understand health information [1]. A health-literate organization is one that actively works to remove barriers by implementing clear communication strategies, training its workforce on health literacy principles, engaging communities in co-designing health services, and embedding health literacy into its leadership priorities [2].

Hispanic/Latine adults have the lowest average health literacy in the United States [3]. Both patients and practitioners underestimate the potential impacts of low health literacy and limited language proficiency [4, 5], which include worse health outcomes, higher medical costs, and avoidable hospitalizations [6]. A 2017 study by Levy and Janke linked health literacy with access to care and suggested that interventions to address health literacy “should look beyond the clinical encounter” [7].

Human-centered design (HCD) is a paradigm that puts the end user at the center of the solution design process [8]. In public health, the HCD methodology can empower historically excluded communities that face structural and cultural barriers [9, 10]; build more resilient systems of care for patients and practitioners [11]; and develop complex, multicomponent interventions [12]. Impediments to the adoption of HCD in health science research and public health activities include cost, time investment, training needs, and a cultural mismatch with the more rigid hierarchies of academic science [10]. The HCD process may require more generous timelines, the integration of key stakeholders into every stage of work, and an understanding that the co-designed solutions may not fully align with the funder’s mandate [13].

Black and colleagues describe a case study in which community-based participatory research (CBPR) activities were augmented with HCD methods to adapt a mental health intervention to a refugee population in the United States. The co-design process yielded a product that reflected the values, perceptions, and expectations of the target population. Notably, the process also exposed biases and knowledge gaps among the research team. However, co-design teams faced time constraints, varying levels of digital literacy among stakeholders, and recruitment and retention problems [9].

A 2017 scoping review of the applications and context of human-centered design in global health found that most studies that reported on HCD design methods did not describe a full project cycle [14]. This paper responds to this identified gap in the literature by describing the results of a local Advancing Health Literacy (AHL) project led by the Pima County Health Department (PCHD), which sought to advance health literacy and adherence to COVID-19 public health practices among Hispanic/Latine adults in Pima County of childbearing age (20 to 45 years old) with the ability to become pregnant.

In 2021, Pima County received a grant from the Advancing Health Literacy to Enhance Equitable Community Responses to COVID-19 initiative (US Department of Health and Human Services Office of Minority Health) [15]. Due to the high national levels of COVID-19 vaccine hesitancy reported among pregnant individuals [16] and Hispanic and Latine individuals [17, 18], PCHD opted to focus its efforts among Hispanic and Latine individuals of child-bearing age with the ability to become pregnant. Pima County proposed to use HCD solution design approaches, which have traditionally been leveraged in global health and international development but are increasingly recognized as valuable in US public health and healthcare innovation [19–21]. Because HCD relies on self-reported insights, the methodology and findings were complemented with a behavioral science approach and lens, which draws upon a deep evidence base describing how to effectively shift important health behaviors [22].

The solution design research process for the AHL project in Pima County yielded community-sourced suggestions for a community pilot intervention to improve health literacy at the individual level and a clinical pilot intervention to improve health literacy at the organizational level. While both pilots are considered together in this paper as two complementary components of the overall effort, no direct statistical comparison was conducted between the two groups, as the focus is on within-group changes rather than between-group differences.

## Human-centered design process

### Solution design: Community-based research process

The AHL project sought to increase the capacity of the health literacy ecosystem of practitioners within Pima County using a knowledge management model known as a Community of Practice (CoP), defined by Wenger and colleagues as a group “of people who share a set of problems, or a passion about a topic, and who deepen their knowledge and expertise in this area by interacting on an ongoing basis” [23]. Originally deployed within the business sector, CoPs have been identified as a promising model for public health that provides opportunities for individuals and organizations to collaborate and grow within a shared domain of interest or practice [24, 25]. The Pima County Advancing Health Literacy CoP included an HCD consultancy, a behavioral science firm, a multisite Federally Qualified Health Center (FQHC), two local community-based organizations (CBOs), Pima County Libraries, and a team of academic evaluators. Over the course of the project, the CoP grew to include organizations that support Community Health Workers (CHWs), known to the target population as *promotoras de salud* (“health promoters”) or *promotoras*.^1^

**Fig. 1:**
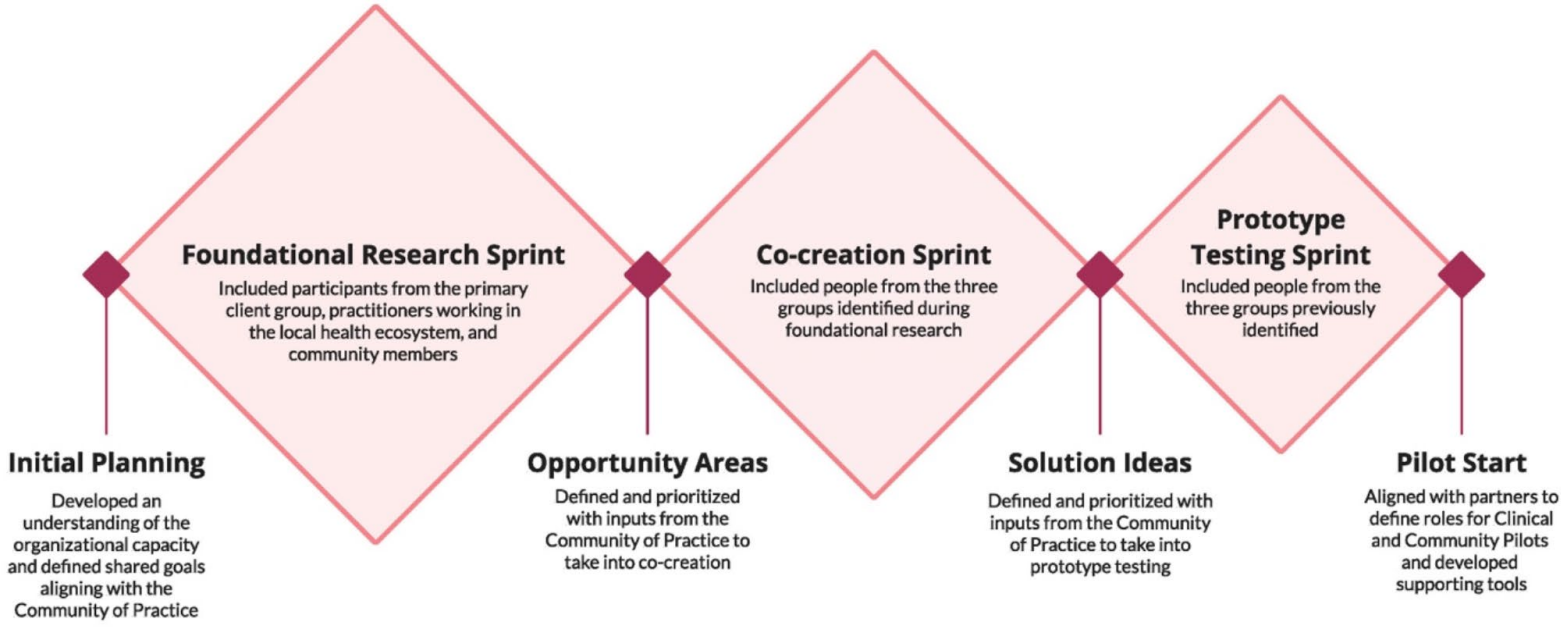
HCD framework of sprint-based process deployed for the AHL project

The health department worked with the design consultancy to facilitate the three phases of the HCD process to yield interventions for piloting and implementation: (1) foundational community-based research, (2) co-creation sprints, and (3) prototype testing sprints (Fig. 1). Because the intervention ideas that resulted from this community-based HCD process were specific to community and clinical settings, the resulting pilot focused on interventions in those two settings. Community efforts focused on building and enhancing health literacy at the personal level to improve access to care within the primary group of focus. Clinical interventions were designed for healthcare practitioners and sought to build trust and understanding between practitioners^2^ and patients from two clinics of a multisite Federally Qualified Health Center (FQHC) in southern Arizona.

The foundational research phase involved 13 practitioner interviews and 8 small group discussions with 32 clients and 9 community members.^3^ Clients were recruited from two clinics within the FQHC with the help of a selection criteria recruitment tracker created by the HCD firm. As the foundational research progressed, clients identified trusted sources of information within their families, friend groups, and community, several of whom were recruited to provide input as community members within the foundational research. Clients and community members were compensated according to an equitable model that will be detailed in a separate publication. The group discussions featured interactive HCD activities to identify common and outlier barriers and facilitators to engaging with the healthcare system, navigating resources, and recognizing trusted information sources, especially in the context of different cultural practices between patients and practitioners, as well as patients and the healthcare information systems they access.

The foundational community-based research phase of the HCD process identified health-seeking behaviors that divided care between health systems in the US and Mexico. Participants reported that, in addition to cultural and linguistic factors, they sought care in Mexico due to decreased wait times for appointments and better access to practitioners. These findings align with prior research documenting similar cross-border health-seeking behaviors among Mexican immigrants in California [27] and broader patterns of healthcare access disparities among Hispanic immigrants in the US [28]. This consistency with prior literature highlights the persistent structural barriers to healthcare access and underscores the need for interventions that improve both individual and organizational health literacy.

### Solution design: Identifying and prioritizing opportunity areas for design

**Fig. 2:**
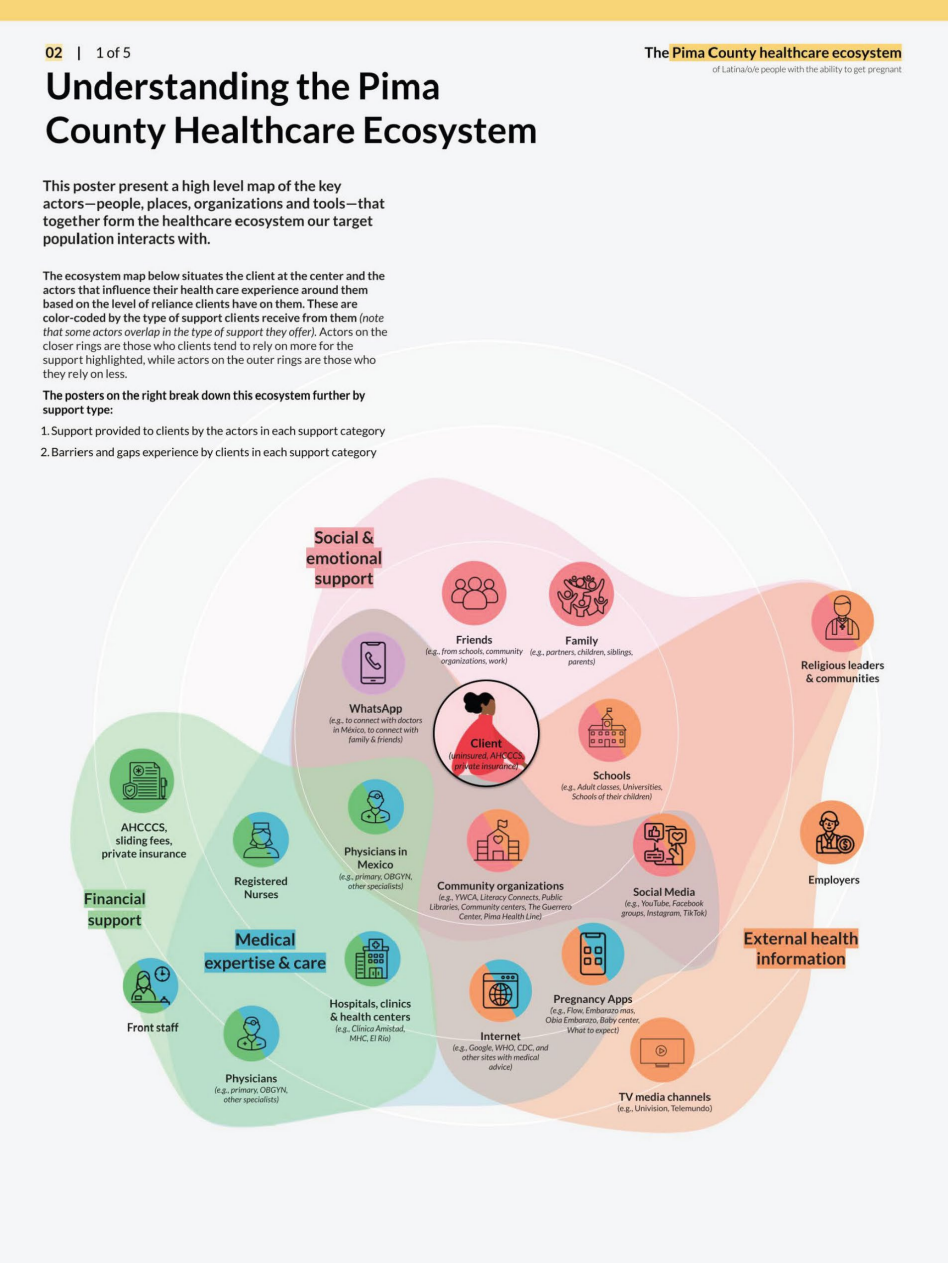
Understanding the Pima County Healthcare Ecosystem

**Fig. 3:**
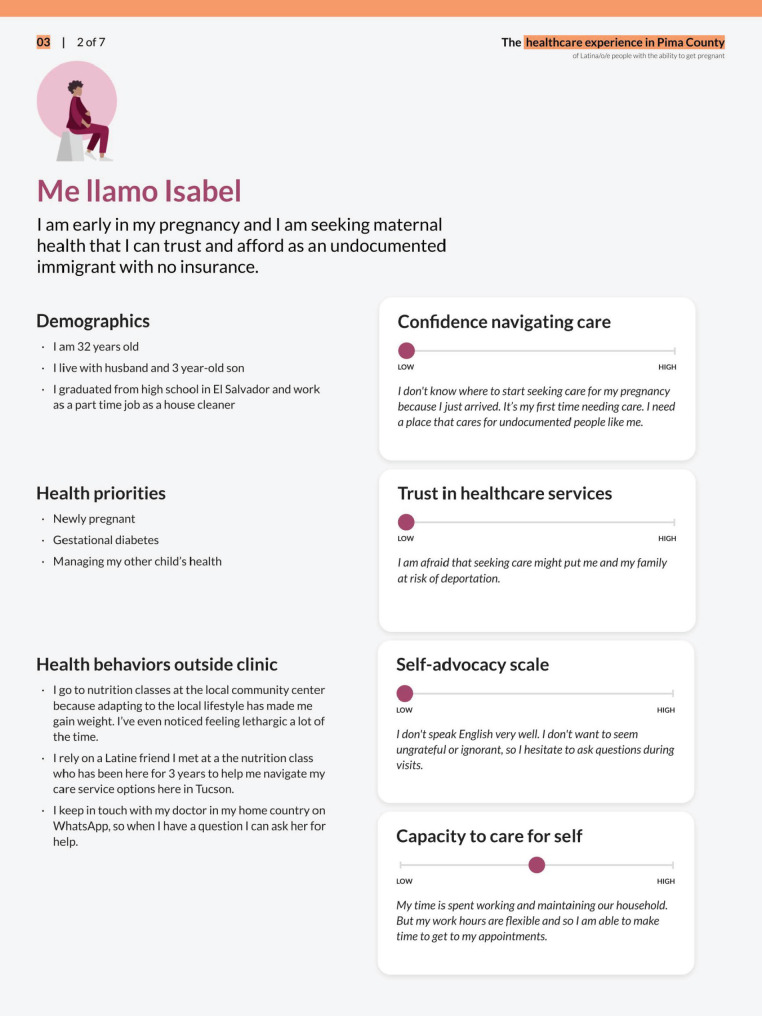
Primary Client Persona

**Fig. 4:**
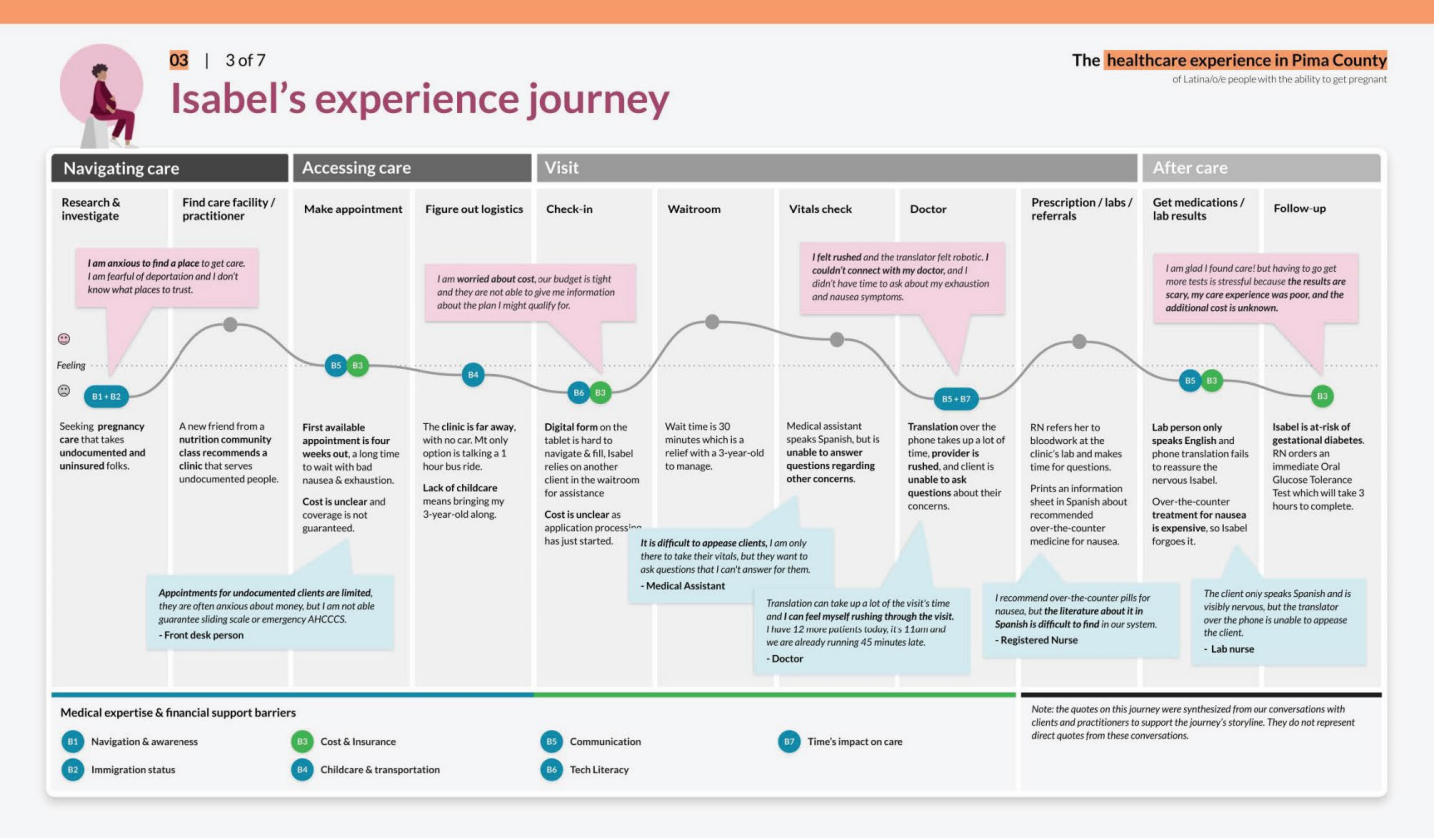
Primary Client Persona Health Seeking Journey

**Fig. 5:**
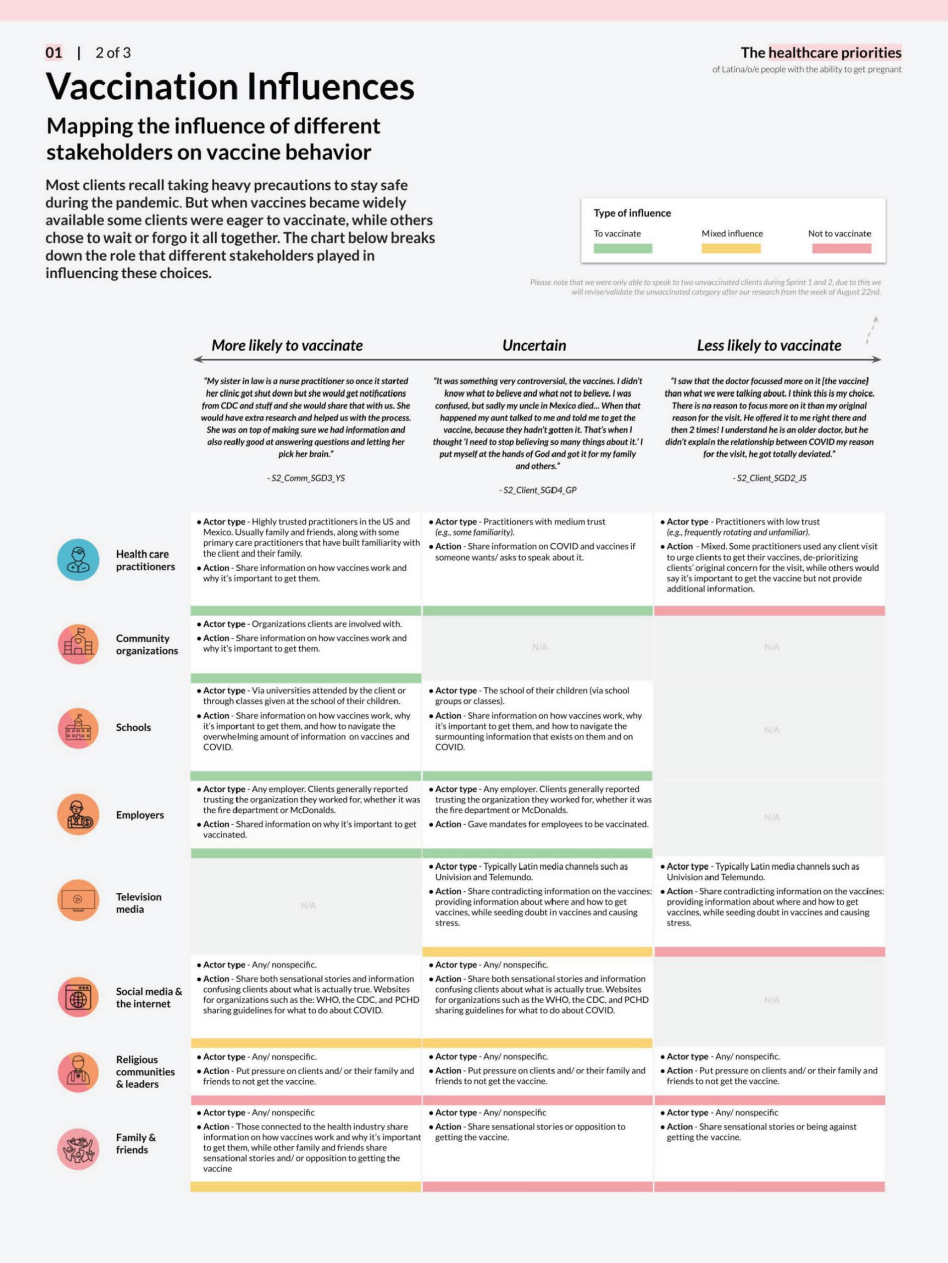
Vaccination Influences Map

Deliverables from the foundational research phase included a map of the health care ecosystem (Fig. 2), user personas (Fig. 3), journey maps (Fig. 4), and influence maps (Fig. 5). These deliverables were reviewed with the CoP to define and prioritize opportunity areas with the most impact potential and those most feasible to pilot within the project performance period. The prioritization process was guided by an asset-based community development approach, which focuses on leveraging existing community strengths and resources to drive sustainable health improvements [29, 30]. Opportunity area decisions also reflected the capacity of CoP members, including governmental and healthcare systems (Additional File 2). Facilitating this work enabled the local health department to redistribute power to the community through inclusive research and the collaborative identification of opportunities for co-design.

### Solution design: Co-creation

After translating the prioritized opportunity areas into a set of relatable scenarios, the design consultancy facilitated a series of co-design sessions with 9 clients, 6 practitioners, and 8 community members to enable them to brainstorm ideas for solutions to the barriers and challenges portrayed in the scenarios. These ideas were synthesized and collected into a portfolio of 9 concepts: (1) a healthcare navigation peer group and class led by a *promotora*, (2) a WhatsApp^4^ chat line and support group, (3) free mobile clinics, (4) health navigation resource guides, (5) a membership network for free and affordable health care, (6) an in-clinic health *promotora*, (7) a Patient Goal Tool to help clients prioritize their health needs before an appointment, (8) clinical visit enhancements to build trust, and (9) a user-friendly redesign of the visit summary complete with prompts and resources for patients’ next steps in their health care journey. To guide the conceptual approach, we applied the EAST framework (Easy, Attractive, Social, and Timely), a widely used behavioral science model designed to encourage behavior change by reducing friction, increasing engagement, and leveraging social norms [32]. This framework informed the development of prototype interventions that were later tested for feasibility and impact.

**Fig. 6:**
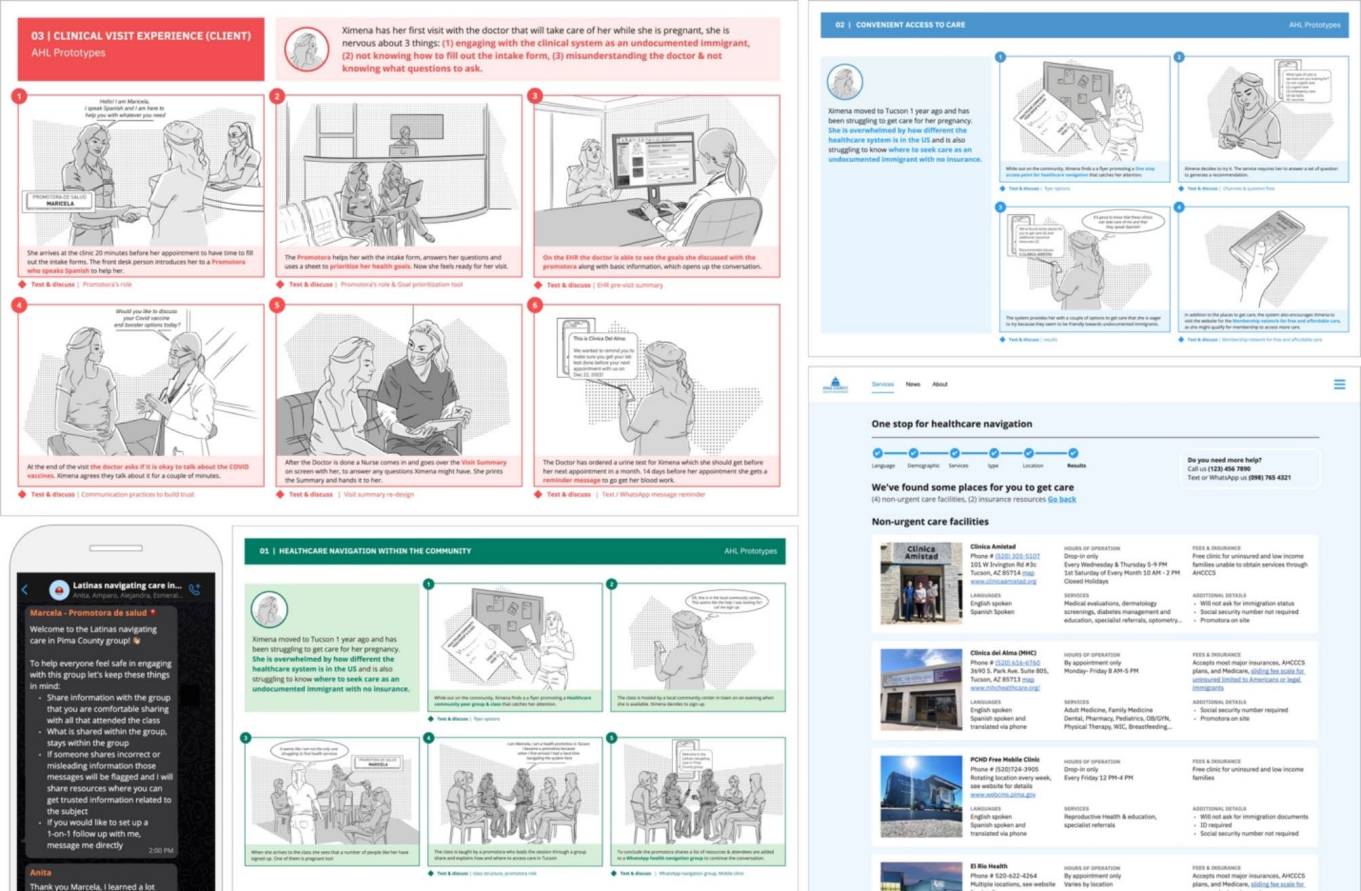
Selected Prototype Testing Sprint Materials

The prototyping phase produced a set of storyboards that translated the proposed prototypes into a set of visual narratives (Fig. 6) for review by clients, practitioners, and community members. With their feedback, the design consultancy, in close collaboration with the CoP, consolidated the most promising and feasible prototypes into a blueprint for a clinical and a community pilot.

The HCD process produced a multicomponent solution involving *promotoras*, community health clinics, practitioners, and community members. Individually, none of the piloted interventions relied on novel or untested theories. In aggregate, however, the design process helped build capacity among participants. Additionally, the potential solutions were validated through an evidence review conducted by the behavioral science firm, which suggested that these types of solutions had worked in similar contexts and therefore, would constitute a strategic and resource-savvy, multi-component approach. For example, several studies have pointed to the effectiveness of the *promotora* model to facilitate culturally appropriate health interventions [33, 34]. This combination of community- and evidence-informed strategies and increased capacity among stakeholders fostered the use of sustainable tools and programming to improve both individual and organizational health literacy.

## Methods

### Community pilot intervention setting

Two intervention ideas from the solution design process were implemented and coordinated by two local CBOs. Both were facilitated primarily in Spanish by *promotoras* and CBO staff. Some English workshops were conducted with translation and interpretive services made available as needed.

- a self-efficacy and health literacy workshop for community members recruited by the CBOs that addressed health care facility types, payment, insurance, appointment preparation, and patient rights.
- a WhatsApp follow-up group for workshop attendees, facilitated by a promotora who shared resources and monitored interactions among participants.

### Clinical pilot intervention setting

**Fig. 7:**
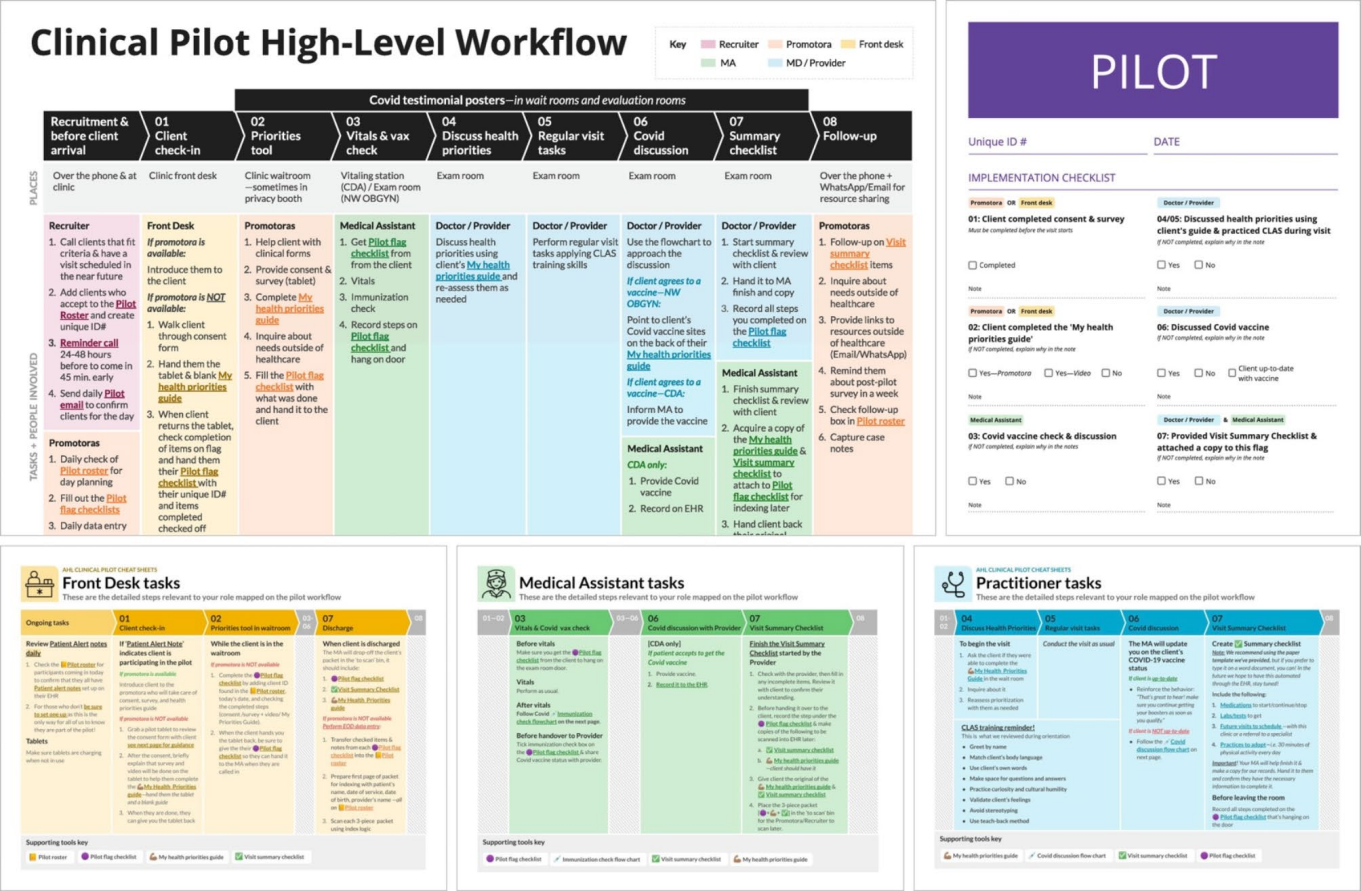
Pilot Implementation Guidelines, Processes, and Training Tools

The clinical pilot intervention took place at two clinic sites within a multisite FQHC, a primary care and an Obstetrics & Gynecology clinic, and incorporated the 7 interventions listed below. Figure 7 represents the pilot implementation workflow for clinical operations and individual practitioners. All materials were provided in English and Spanish.

- A pre-visit appointment with the promotora to support the client as they completed a Patient Goal Tool which helped the patient prioritize their health concerns in the appropriate language and cultural context, encouraged shared-decision making and supported patient-centered care [35].
- A Pilot Flag Checklist that helped practitioners support the different clinical pilot interventions (Fig. 7).
- Practitioner training on how to approach conversations with patients about COVID-19 vaccines.
- Culturally and Linguistically Appropriate Services (CLAS) training for practitioners that included a training on how to deliver the clinical interventions.
- An After-Visit Checklist for patients that summarized the next steps in their healthcare journey and provided an opportunity for employing the teach back method [36].
- Clinic marketing materials to prompt practitioners and patients to talk about COVID-19.
- A *promotora* follow-up with patients a few weeks after their visit to offer support with next steps.

### Evaluation

Community and clinical pilots were evaluated through a mixed-methods approach. For both pilot projects, participants were asked to complete pre- and post- surveys (Additional Files 4, 5) that estimated the effect of an intervention by comparing outcomes before and after implementation. The pre- and post-surveys assessed the following:

- Changes in health-seeking behaviors.
- Barriers to health-seeking behaviors.
- Participants’ experiences with the medical team.
- Vaccination status.
- Health literacy.
- Where participants hear or learn about vaccination information.
- Participants’ satisfaction with the interventions.

Sub-components of the surveys addressed changes in both COVID-19 immunization and health literacy.

Changes in COVID-19 vaccination status were assessed through survey questions probing participants’ self-reported vaccination status before and after the interventions. Specifically, participants were asked whether they were up to date on their COVID-19 immunization series, needed a booster, were unvaccinated, or were unsure.

The health literacy scale used for both pilots was adapted from the BRIEF Health Literacy Screening Tool to measure (1) how often someone helped the participant read materials related to their health, (2) how often the participant had problems learning about their medical condition because of struggles to understand written information, (3) how often the participant had a problem understanding what was told to them about their health, (4) the participant’s confidence in filling out medical forms by themselves.

Quantitative data analysis compared numerical variables using a Paired t-test and categorical variables were analyzed using Chi-square tests. For variables that were exclusive to one of the two surveys, descriptive statistics (frequencies and averages) were calculated to summarize their distribution.

Qualitative methods included structured interviews with pilot participants randomly selected on the basis of their initial consent and willingness to participate in the interview process. Qualitative Rapid Analysis (RA) was employed post-interviews by two qualitative coders [37]. The thematic (RA) analysis process involved developing a template summary table the team could populate with the data extracted from the interview transcripts, including illustrative quotes, and researcher’s notes. The initial themes were based on the human-centered design framework and the identified topics by the community stakeholders during the co-design processes but allowed for the flexibility of identifying and adding new themes that emerged based on the interviews [38]. One of the benefits of utilizing rapid analysis is that the coders assessed data saturation as the summary table was completed for each interview that was conducted. The resulting themes illuminated insights from both the community and clinical pilots, addressing aspects such as health literacy and access to healthcare.

## Results

### Community pilot results

Of the 258 community members who met the inclusion criteria of the community pilot, 207 completed pre- and post-surveys. Almost 50% were between 36 and 45 years old with 16% pregnant at the time of the workshop.

On average, participants reported a 10% increase (0.25% points) in confidence in health-seeking behaviors (Table 1). This difference in confidence (rated on a scale of 1–3) before, and 8 weeks after, the workshop was statistically significant. Participants reported higher increases (more than 15% points) in confidence for several key health-seeking behaviors and topics that were covered in the workshop.

**Table 1:** Confidence in health-seeking behaviors before and after community pilot

Health-Seeking Behavior	Community Pilot	
	Pre-survey (“I can do this already”)	Post-survey (“I can do this already”)
Book or schedule a healthcare appointment	66.2%	85.5%
Access community resources and tools like health events, classes, and support groups	29.5%	58%
Find a COVID-19 vaccine or booster	65.7%	83.6%
Discuss my health needs with my medical team	57.5%	77.8%
Find useful information and resources for my health	37.2%	69.1%
Ask for an interpreter if needed	54.1%	76.3%
Complete medical forms	71.5%	85%
Ask for a doctor or nurse who shares an identity with me	49.3%	68.6%
When and how to start or stop taking medicine	61.4%	80.7%
Talk to a trusted family member, friend, or community member about my health	65.7%	84.1%

Participants reported a statistically significant difference in the average number of barriers experienced before and after the workshop. The decreases ranged between 1% and 12% by barrier (an average of 0.59% points). The decrease was greater for the topics covered by the workshop. There was no statistically significant difference in health literacy or COVID-19 vaccine uptake.

Qualitative data were collected by the evaluation team through structured interviews with participants (*n* = 17), promotoras (*n* = 2), and workshop and WhatsApp group facilitators (*n* = 4). The following themes emerged from these interviews:

- Understanding healthcare navigation.
- Communication with healthcare practitioners.
- Empowerment through knowledge.
- Undocumented status.
- Access to healthcare.
- The impact of the workshop and WhatsApp group.

Illustrative quotes from community pilot participants are listed in Table 2. Qualitative data support the quantitative findings of increased confidence in health-seeking behaviors and accessing culturally responsive care. An example of this is found in a participant sharing, “I encountered situations where nurses sometimes did not want to speak Spanish… Now I know that for me it was my right to ask a nurse to help me in Spanish.”

**Table 2:** Themes and illustrative quotes from the community pilot study

Interview Group	Theme	Illustrative Quotes
Community Pilot Participant	Understanding Healthcare Navigation	“The biggest thing that I learned was the difference between emergency versus urgent care… It’s kind of hard to tell where to go.” (Participant 1) “I’m grateful that I was able to take this workshop because it gave me the opportunity to know that if they told me one thing, it was not the only option, that I had the right to ask and say more things…” (Participant 5)
	Communication with Healthcare Practitioners	“But I also think that we as patients, if they [we] don’t see an interest in our own health, then neither do they [the practitioners].” (Participant 8) “I encourage myself to go deeper, to ask more… I feel more confident about making changes.” (Participant 7)
	Empowerment through Knowledge	“Having knowledge opens doors for you. It helps you change your mentality to know where to go and well, when we don’t have all this information that you are providing [in the community workshop], even if we ask a neighbor, a friend, an acquaintance.” (Participant 6)
	Undocumented Status and Access to Healthcare	“I do have friends that are trying to get their green card, and they don’t know where to go. They think. ‘I can’t get medical help for me or my children because of my status.’ And I’m like, ‘No, no, you can!” (Participant 2) “When I arrived to the US I was four months pregnant and they didn’t treat me until I was like five months. I didn’t know I could get treatment… I loved it [the workshop] because it gives you a lot of information so that you are informed about what option[s] you have to treat you.” (Participant 4) “To me it seemed super important of the rights that any person can have… they have the right, it is a right to receive medical care and that they [any person] should not be treated differently, especially due to immigration status.” (Participant 7)
	WhatsApp Group Chats	“I chose to join the WhatsApp group. The truth is that I also liked it a lot… you receive very, very valuable information. Information about the clinics, programs or activities… We are also given support if we have any questions.” (Participant 4) “Being in the WhatsApp group… it was a good resource and it was good to know that I had that support, that if one day I had a question or a doubt, or if I knew something I knew where to go look for that.” (Participant 5)
Facilitator	Vision for the Future	“I would like them [the community] to have that information and for it to be more open to the general population, to everyone so that everyone has access to that information [navigating healthcare].” (CBO Facilitator 1) “I would like to see this continue on in the community. I think people benefited from this workshop.” (CBO Facilitator 2)

### Clinical pilot results

Of the 95 clients who participated in the clinical pilot, 3 did not complete the pre- and post-survey. Results indicated that the interventions increased clients’ confidence in health-seeking behaviors. On average, participants reported a 6% increase (0.28% points) in confidence in health-seeking behaviors (Table 3). This difference in confidence (rated on a scale of 1–6) was statistically significant. The health behaviors with the greatest increases in confidence (Table 3) were in:

- Asking the health care team for additional resources.
- Asking the health care team questions.
- Discussing health needs.
- Booking health care appointments.

**Table 3:** Confidence in health-seeking behaviors before and after clinical pilot

Health Seeking Behavior	Pre-survey (“I do this already”)	Post-survey (“I do this already”)
Ask my healthcare team questions	51.1%	69.6%
Ask my healthcare team for additional resources and tools	28.3%	51.1%
Book or schedule a healthcare appointment with my current provider	63%	72.8%
Book or schedule a healthcare appointment with a new provider	27.2%	48.9%
Book or schedule a healthcare appointment with a specialist	26.1%	44.6%
Book or schedule a lab appointment for something like a blood draw or special test	41.3%	55.4%
Book or schedule a COVID-19 vaccine appointment	26.1%	26.1%
Access community resources and tools for COVID-19 vaccines and boosters like free mobile clinics	13%	26.1%
Access community resources and tools like health event classes, and support groups	13%	23.9%
Find information and resources for my health	43.5%	58.7%
Find information and resources for COVID-19 vaccines and boosters	26.1%	31.5%
Find a COVID-19 vaccine site	26.1%	33.7%
Discuss COVID-19 vaccines with my healthcare team	28.3%	37%
Discuss my health needs with my medical team	46.7%	64.1%
Fill a prescription	52.2%	54.3%
Follow the instructions on the label of a medication bottle	65.2%	70.7%
Start or stop taking medication based on a doctor’s or pharmacist’s direction/recommendation	51.1%	58.7%
Complete medical forms	48.9%	55.4%
Review my next steps and actions items after an appointment	44.6%	54.3%
Talk to a trusted family member, friend, or community member about my health	57.6%	60.9%

Overall, participants rated themselves as having greater health literacy after the interventions. The number of participants with “marginal” health literacy increased by 15% points, while those with “limited” health literacy decreased by 11% points. Clients also reported fewer perceived barriers to achieving health, such as less confusion and fewer language barriers in the healthcare system.

Qualitative interviews with 15 clients, 10 practitioners, and 2 promotoras from the clinical pilot identified these themes, which are listed in Table 4 with illustrative quotes:

- Benefits of interacting with a *promotora* (community health worker).
- Future considerations from the practitioner’s perspective.
- Changes in interactions with healthcare practitioners, including the importance of comprehensive follow-up mechanisms and participant engagement strategies.
- The benefits of the Patient Goal Tool in facilitating individualized care, aiding memory, and fostering confidence in communication with healthcare practitioners.

**Table 4:** Themes and illustrative quotes from the clinical pilot study

Interview Group	Theme	Illustrative Quotes
Clinical Participants	Changes in Interaction with Healthcare Practitioners	“If they don’t have the answer, they give me more. They don’t leave me halfway. They help me complete what I need like references to other places if they [healthcare practitioner] can’t help me.” (Participant 2)
	Benefits of the Patient Goal Tool	“I think it was definitely like a help because sometimes you have in your mind like questions that you want to make your doctors, but you don’t always remember them during the appointment…” (Participant 10) “…I feel like it helped because now I…prioritize like what I need to ask…” (Participant 7)
	Interaction with the Promotora (Community Health Worker)	“Well, more than anything, they [the promotoras] clarified the doubts.… I should focus on what to ask, any question, That’s what the doctors were for: to serve me, to help me.” (Participant 1)
Practitioner	Future Considerations	CHW in Clinic: “I think having a promotora [CHW] and having somebody here that could help with filling out papers, help with setting goals for the patients, that would be great.” (Practitioner 1) “Having somebody like that, that can be almost like not like a social worker, but almost to help with community outreach?… We don’t have that, and we need that. " (Practitioner 2) CLAS Trainings: “To have a really truly successful and culturally competent center, it’s important for everyone to get access to more learning and education.” (Practitioner 3) “I think it would be really helpful for everyone that we work with to have access to the trainings, including front desk people, MAs, everyone.” (Practitioner 4)

Abbreviations: CHW, community health worker. CLAS, culturally and linguistically appropriate services

Due to the nature of the pre/post quantitative process evaluation, these findings cannot be used to determine causality. Future research should employ more rigorous study designs, such as randomized controlled trials, to evaluate the validity and causal mechanisms underlying these results.

## Discussion

### Community pilot

The community pilot was a co-designed solution that focused on access to health care as a way to offset the health impact of low health literacy. The community pilot participants’ health literacy was not improved by the interventions. More specific and targeted interventions may have resulted in a shift in health literacy indicators such as understanding health information and confidence filling out medical forms. To achieve measurable change, future workshops should be offered in a series and consider allowing participants multiple opportunities to practice the concepts through interactive materials and real-world examples. This approach may offer participants more knowledge and confidence to fill out medical forms or prepare questions prior to their appointments.

The community pilot also revealed no statistically significant difference in COVID-19 vaccine uptake. The community interventions were not focused on vaccination; at the time of pilot implementation, the federal public health emergency declaration had expired, tolerance for conversations about COVID-19 vaccines was waning, and the majority of the participants were already vaccinated.

We observed statistically significant increases in health-seeking behaviors, such as finding and accessing resources and discussing health needs. These results suggest that the community pilot bolstered participants’ health agency, which is critical for building long-term health literacy. Throughout the interview process, participants, facilitators, and partners expressed their satisfaction with the health literacy workshop and hoped it would continue beyond the pilot timeline. One participant noted: “If my mother had had access to this workshop, it would have positively changed her life. She never went to the dentist and only ever had emergency dental work. I didn’t know that there were places we could have taken her for routine dental work.”

While health literacy was not improved by the community pilot interventions, the increase in confidence and perceived decrease in barriers to health seeking behaviors suggest that health literacy is just one (albeit important) factor in achieving healthier outcomes. Participants and community partners spoke of the need for better access to health care and health information. Community partners saw health care as a universal right that could only be accessed if people understood how to advocate for their own health rights, regardless of their legal or immigration status. Participants wished for a relationship with their US doctors that resembled the prompt and responsive care they received in Mexico, echoing findings from Bergmark and colleagues [39], who found that some Mexican immigrants preferred Mexican healthcare practitioners because of their emphasis on clinical discretion and more personalized care, as well as those of Horton and Cole’s 2011 study which found that Mexican immigrants often described US healthcare as overly bureaucratic and rushed compared to the more attentive care they received in Mexico [40].

Our study’s findings mirror Stormacq and colleagues’ 2020 systematic review recommendations that future health literacy interventions must be culturally appropriate or group-specific, while being person-centered [41]. This makes it possible to consider both the context and the characteristics at the community and the individual levels.

### Clinical pilot

Health literacy increased among clients after the clinical interventions. This may be related to the following factors, which distinguished the clinical pilot from the community pilot: (1) Participants within the clinical pilot were already interacting with a medical system and received more interventions than community pilot participants; (2) practitioners were trained in culturally and linguistically appropriate service (CLAS) standards of care, allowing a proactive approach to address patients’ challenges in understanding their health condition(s); and (3) the one-on-one nature of the clinical pilot interactions provided greater individual customization than the group setting of the community pilot.

The HCD process revealed that many of the patients who were more accustomed to receiving healthcare in Mexico felt that the rushed nature of their appointments in the US precluded them from asking questions, a finding that is emphasized in numerous studies that examine the lack of *personalismo* (warm, caring, and trusting interactions in healthcare settings) and language-concordant care as contributing factors to the so-called Latino Health Paradox, which highlights the greater life expectancy of Hispanic and Latine individuals over non-Hispanic white individuals despite their lower socio-economic status, and posits that as healthy Hispanic and Latine individuals increase their acculturation in the US, their access to factors that protect their health, such as traditional dietary patterns, culturally tailored health interventions, and trust in their healthcare practitioners, may diminish, along with their health [42–44]. The combination of the *promotora*-supported pre-visit appointment with the completion of the Patient Goal Tool provided clients with a framework for their clinical visit before it began, and a practice they could replicate for future visits to maximize the value of their limited time with their practitioners. The Patient Goal Tool also allowed practitioners and clients to work together to assess whether additional appointments would be necessary, and it informed the tailored creation of the After-Visit Checklist. Together, these tools and practices increased the number of opportunities for individualized and culturally relevant, shared decision making between clients and practitioners, increasing the opportunity for patient-centered care. The findings of the present study speak to the recommendations set forth by Walters and colleagues’ 2020 systematic health literacy review observing that there was not enough focus on patient-centered outcomes, and interventions could be more useful if they involved patients in the design rather than assuming that simply telling people what they need to do is sufficient to bring about change [45].

Eighteen *promotoras* were provided with 20 h of core competency training leading to the certification of 3 *promotoras* by the end of the grant award. Their value and capacity proved a surprise even to themselves, with one *promotora* commenting in a post-intervention interview, “It was a dream come true to get certified as a CHW through this pilot program. I didn’t know that I was one before.” Due to their unique position as a patient guide and advocate, the *promotoras* influenced the refinement and sustainability of the interventions beyond the clinical pilot period. At a meeting on May 21, 2024, a representative of the multisite FQHC represented on the authorship team indicated that their organization had since hired five full time *promotoras*, honoring a long tradition of *promotoras* as agents of social change [46].

### Study limitations

Unintentional bias existed in the sample recruitment for both pilots. Participants for the community pilot were recruited through flyers posted in the community spaces across Pima County and via the community partners’ websites. The sample may not be representative of all Pima County residents because of self-selection: People who signed up were already likely to engage in community-based activities and/or were already interested in healthcare topics. The clients of the clinical pilot were patients with existing relationships with the FQHC practitioners.

The time frame created a limitation. This challenge was exacerbated by the number of interventions involved in the pilot, the narrow inclusion criteria for participants, and most significantly, the compressed time frame between administration of the interventions and the post-survey. Evaluators would have preferred a longer time frame to observe potential behavioral changes that were longer-term rather than short-term, and to probe the lasting significance of the interventions on participants’ ongoing health-seeking behaviors.

## Conclusion

Shifting health literacy at the individual and organizational levels requires commitment, time, and flexibility. This study found that human-centered design and behavioral science can identify promising multicomponent solutions that prioritize the voices and honor the ideas of a historically and contemporarily excluded group. The innovative design methods developed a comprehensive picture of the health ecosystem and the behavioral opportunities to improve health literacy and access to care. Further efforts in this area should prioritize robust networks of research and implementation that center individuals and the organizations that serve them at every stage of work.

## Electronic supplementary material

Below is the link to the electronic supplementary material.

Supplementary Material 1

Supplementary Material 2

Supplementary Material 3

Supplementary Material 4

Supplementary Material 5

Supplementary Material 6

Supplementary Material 7

## Data Availability

The data and materials from the HCD process and pilot evaluations are available from the corresponding author on reasonable request. The pilot evaluation (both quantitative and qualitative) data are available in a de-identified and aggregated form.

## Footnotes

1 The *promotora de salud* model originated in Central and South America and has a long history of community-based support to improve health outcomes through social connectedness and improved linkages to care. Promotoras serve as vital links to their communities and health and social service practitioners that have been impacting Arizona communities for over 50 years as frontline public health workers. Many live within the communities they serve or have a deep understanding of the community and its needs, making them trusted sources of information [26].

2 In this paper, the term “practitioners” refers to individuals employed by the FQHC who were actively involved in the clinical pilot, including physicians (MDs and DOs), nurse practitioners, midwives, medical assistants, managers, and front office staff.

3 In this paper, the term “clients” refers to individuals involved in the foundational research who received services through the participating FQHC’s clinics. The term “community members” refers to others involved in the foundational research, often from within the clients’ sphere of influence, who were not receiving clinical services through this organization. “Participants” refers to clients and community members involved in the respective clinical and community intervention piloting phase. The participants involved in either the clinical or community pilots were kept separate so as not to adversely impact evaluation. As a result, clients of the FQHC were not recruited to the community pilot or vice versa. This decision was made for evaluation fidelity, despite the belief among designers and program staff that the two sets of interventions could increase health literacy at an even greater rate if participants could benefit from both.

4 WhatsApp is a closed-network social media platform used by Hispanic/Latine adults [31].

## Declarations

Key elements of the consent form included:

 • A summary of the research, highlighting the study’s purpose to improve outreach and engagement with healthcare practitioners and Hispanic/Latine/Latinx individuals of childbearing age (20–45 years old) to increase vaccine confidence and coverage of COVID-19 vaccination.

 • A description of the major requirements of the study, including practitioner training, completion of pre- and post-surveys, participant engagement with promotoras, and optional participation in a 1-hour interview.

 • Information on the voluntary nature of participation, emphasizing that participants could withdraw at any time without any consequences.

 • Detailed explanations of the time commitment involved, potential risks and benefits, and the confidentiality measures in place to protect participant data.

 • Contact information for the Principal Investigators and the University of Arizona Institutional Review Board for participants to ask questions or raise concerns.

 To ensure comprehension, consent forms were written in clear, plain language, and were available in multiple languages to accommodate non-English speaking participants. All facilitators, practitioners, and staff who interacted with any program participants were required to take: Human Subjects Research (HSR) content is organized into two tracks: Biomedical (Biomed) and Social-Behavioral-Educational (SBE). Participants provided their consent by signing the consent form, and receiving a copy of the consent form, indicating their voluntary agreement to participate in the study. Participants were informed that their participation was voluntary and that they could withdraw from the study at any time without any consequences to their access to services or other benefits. Confidentiality of all personal information was maintained throughout the study,

## Abbreviations

AHL: Advancing health literacy
CBO: Community-based organization
CBPR: Community-based participatory research
CHW: Community health worker
CLAS: Culturally and linguistically appropriate services
CoP: Community of practice
HER: Electronic health record
FQHC: Federally Qualified Health Center
HCD: Human-centered design
MHC: Marana Health Center
PCHD: Pima County Health Department
RA: Rapid Analysis
