# Supplementary material for: Using human-centered design to advance health literacy in local health department programming: a case study
Source: BMC Public Health. 2025 Mar 31;25:1207. doi: 10.1186/s12889-025-22491-z (PMC11956235; doi:10.1186/s12889-025-22491-z)
Supplement: Supplementary file 1 — Supplementary Material 1 [file 12889_2025_22491_MOESM1_ESM.pdf]

AHL CLINICAL PILOT CHEAT SHEETS

Practitioner Covid discussion flowchart

This flowchart reframes the conversation about Covid vaccines using tested and studied methods

Start here

If the patient has NOT received a Covid vaccine, rely on the 'Key question framework'

"It looks like you aren't up to date on the Covid vaccine. ***Would you like to discuss your options today?"***

OR

If the patient is due for a booster, rely on the 'Presumptive receptiveness framework'

"It looks like you are due for a Covid vaccine booster, ***we can take care of that today"***

Yes, would like the vaccine

Ask MA to provide the vaccine and document it on the EHR

Undecided, leaning in

Make space for an open conversation:

- Ask if the patient has specific questions & answer them using simple language
- Explain why the vaccine is important and how it works, using proven & tested examples:
  a. *"The Covid vaccines work by building up your body's immune system."*
  b. *"They are very safe and work very well at preventing serious illness."*
  c. *"People who have not been vaccinated are 11 times more likely to die from Covid than people who have been vaccinated."*
  d. *"You might still get Covid, but the symptoms will be less severe and unlikely to cause lasting impact to your health."*
- Provide reassurance and evidence:
  a. *"They have been tested in over 100,000 people and over 4 billion people have received them."*
  b. *(If true that you and family have been vaccinated) "I have been vaccinated, my family members have been vaccinated and I recommend that you should get vaccinated too."*
- Discuss potential side effects
- If patient needs time to decide, provide further trusted resources

No, rejects the vaccine

Avoid passing judgement & preserve the real and perceived choice at stake:

"Of course, this is your decision to make. I'm happy to discuss your options and my recommendation but I'll take your lead" and only continue the conversation if the client is open to it

Tested through our Human-centered design activities and citing examples from the following study: "Focus Group Guides for PROmotion of COvid-19 VA(X)ccination in the Emergency Department (PROCOVAXED) Study" https://tools.niehs.nih.gov/dr2/index.cfm/resource/24262
