# Supplementary material for: Using human-centered design to advance health literacy in local health department programming: a case study
Source: BMC Public Health. 2025 Mar 31;25:1207. doi: 10.1186/s12889-025-22491-z (PMC11956235; doi:10.1186/s12889-025-22491-z)
Supplement: Supplementary file 2 — Supplementary Material 2 [file 12889_2025_22491_MOESM2_ESM.docx]

Opportunities Identified and Prioritized During the Two-Day Workshop for the Co-creation Workshops

| Opportunity Category | Opportunity | Intervention Piloted |
| --- | --- | --- |
| Navigating Care | Support clients in navigating and understanding the parts of the system they need to tap into to get care without expecting them to become technical experts.* | Clinical Pilot:   - Promotora pre-visit appointment & Follow-up Conversation - CLAS Training for Practitioners (including *promotoras*)   Community Pilot:   - Health Literacy Workshop - WhatsApp Follow-up Support Group |
|  | Better contextualize support tools and guidance so that they feel culturally relevant to a diverse Latina/o/e client community. | NA |
|  | Provide practitioners with the knowledge and tools they need to provide cost transparency and resource and referral navigation support to clients ahead and after visits. | NA |
| Client-Practitioner Interaction | Shift the interactions with clients from transactional (which is dictated by the system) to relationship building in order to build trust within the limited time available.* | Clinical Pilot:   - CLAS Training for Practitioners - Patient Goal Tool - After Visit Summary Checklist |
|  | Provide practitioners with opportunities to empathize and connect with their client’s cultural background.* | *This opportunity was not prioritized during co-creation sessions with participants, including clients, practitioners and community members.* |
|  | Create lower stakes opportunities to help clients engage regularly with the health system to build their confidence, familiarity and literacy. | NA |
|  | Help practitioners avoid judgment and instead create space to understand the relevance of COVID vaccination to each client’s health goals and burdens | NA |
| Systems and Data Collection | Ensure that learnings about clients’ personal situations, needs, and goals are captured and maintained across visits.* | *This opportunity was not prioritized during co-creation sessions with participants, including clients, practitioners and community members.* |
| Community Support | Celebrate clients’ unique culture to support lifestyle change and health literacy around areas like nutrition, activity, and other daily habits.* | *This opportunity was not prioritized during co-creation sessions with participants, including clients, practitioners and community members.* |
|  | Empower trusted actors (e.g. peer-mothers, CBOs, schools) to serve as reliable, relevant, and trusted sources of information that can help clients navigate the health care landscape while supporting their health decisions.* | Community Pilot:   - Health Literacy Workshop - WhatsApp Follow-up Group   Clinical Pilot:  Based on the results of testing the COVID conversation approach with users, we designed a set of touchpoints designed to build the trust necessary for clients to be receptive to the COVID conversation with practitioners.   - Waiting Room Testimonial Posters - Practitioner COVID Conversation Guide for initial check-in with medical Assistant - Practitioner COVID Conversation Guide |
|  | Employ clients' trust and reliance on the community to improve health literacy and strengthen their ability to advocate for themselves within the health system. | NA |
|  | Inspire and help clients make informed decisions about getting/not getting the COVID vaccines without adding to the ‘information overload’ problem | NA |
| Workplace Culture | Support practitioners in balancing multiple clients and the multitude of challenges they each face within the limited time available without adding to their daily workload and stress. | NA |
|  | Reflect the community's diverse cultural background at health centers by exploring pathways that attract and cultivate more practitioners from the community. | NA |

Abbreviations: CBO, community-based organization. CLAS, culturally and linguistically appropriate services
