# Supplementary material for: Using human-centered design to advance health literacy in local health department programming: a case study
Source: BMC Public Health. 2025 Mar 31;25:1207. doi: 10.1186/s12889-025-22491-z (PMC11956235; doi:10.1186/s12889-025-22491-z)
Supplement: Supplementary file 3 — Supplementary Material 3 [file 12889_2025_22491_MOESM3_ESM.pdf]

03 | NAVIGATING THE HEALTHCARE SYSTEM

PATIENT | COMMUNITY

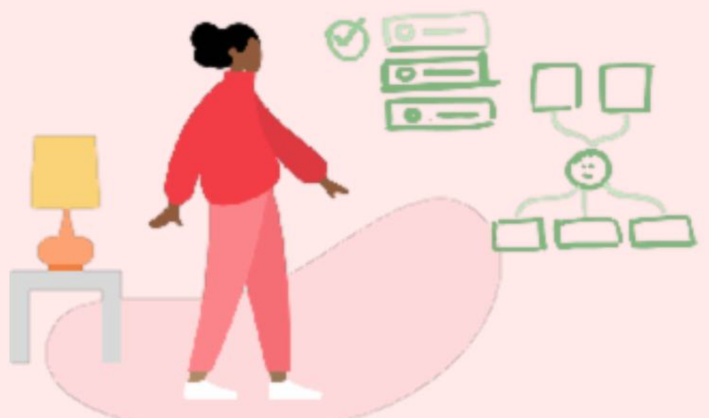

Ximena moved to Tucson 1 year ago and has been struggling to take care of a number of personal health challenges. **She is overwhelmed by how different the healthcare system is in the US** and is also struggling to know **where to seek care as an undocumented immigrant with no insurance.**

DESIGN CHALLENGE

Help Ximena **navigate and understand where and how** she get the care she n[illegible]

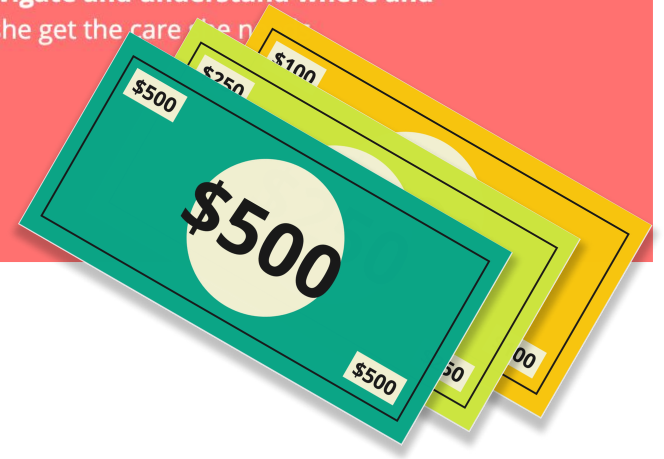

13 | CULTURALLY RELEVANT HEALTH SUPPORT

PATIENT | COMMUNITY

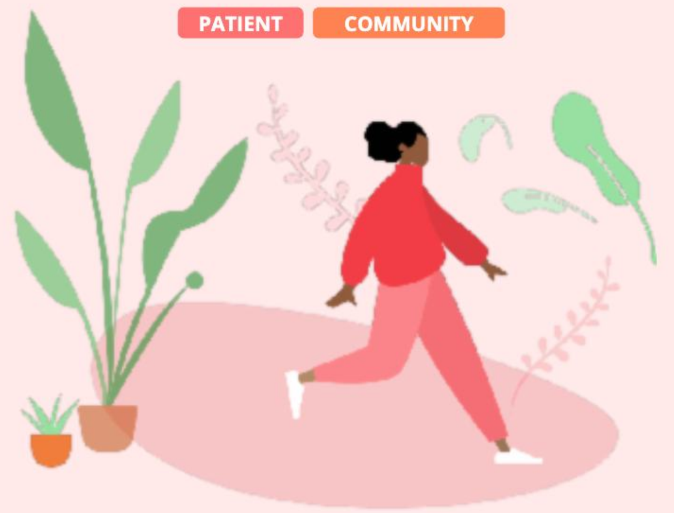

Ximena is pre-diabetic, and **her doctor has recommended she make lifestyle changes related to nutrition and physical activity**. The pamphlet she got at the doctor's office is generic and **she is having trouble applying these recommendations to her daily life**. Ximena knows it will be challenging, but she doesn't want to develop diabetes.

DESIGN CHALLENGE

Help Ximena better **understand her health and get support that considers her culture and situation** so she can successfully make the lifestyle changes she nee[illegible] to make.

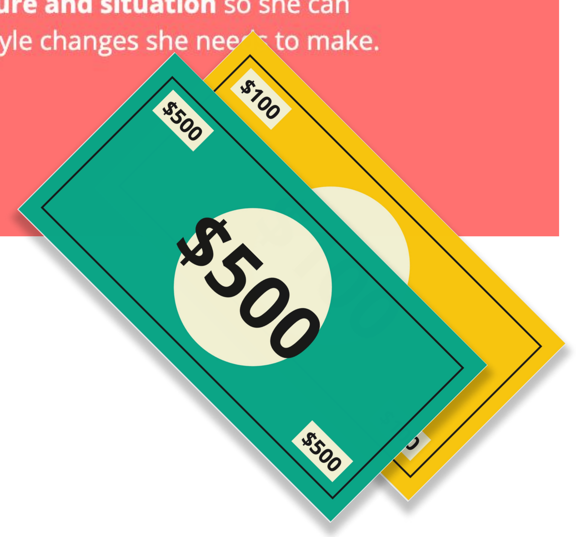

14 | TRUSTED PEERS' ROLE IN NAVIGATING HEALTH INFORMATION

PATIENT | COMMUNITY

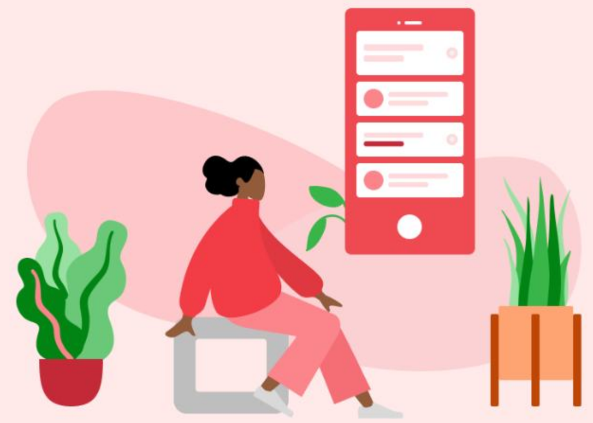

Ximena **feels confused and overwhelmed with all the different stories and information** she hears about global health issues like COVID, Monkeypox, etc. She finds it hard to make her own decisions on these big issues because **she doesn't know who to trust with this information.**

DESIGN CHALLENGE

Help Ximena **know how she can approach and make sense all this information, so she can make her own decision.**

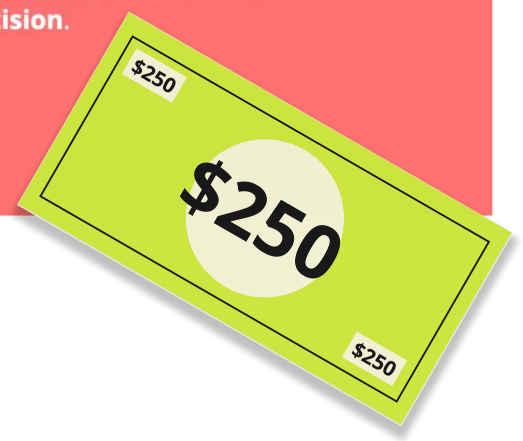

05 | BUILDING TRUST & A RELATIONSHIP WITH PATIENTS

PRACTITIONER

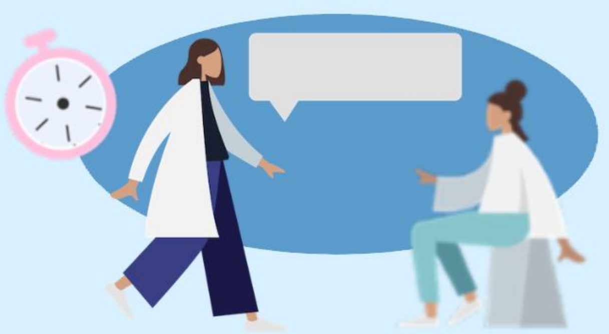

Dr. Smith is **overburdened with a large number of patients** she must tend to daily. She cares about helping her Latine patients but frequently **struggles to build trust and a relationship with them** within the **15 minutes** she has per visit.

DESIGN CHALLENGE

Help Dr. Smith **build trust and a relationship** with her **Latino/a/e patients within the limited time** available

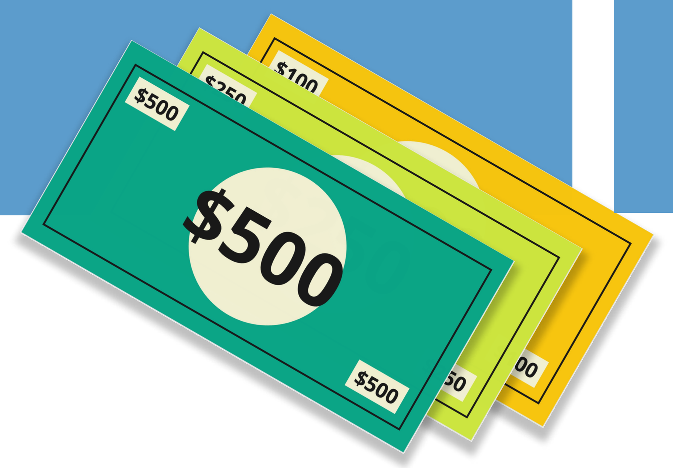

07 | BRIDGING THE CULTURAL DIVIDE

PRACTITIONER | COMMUNITY

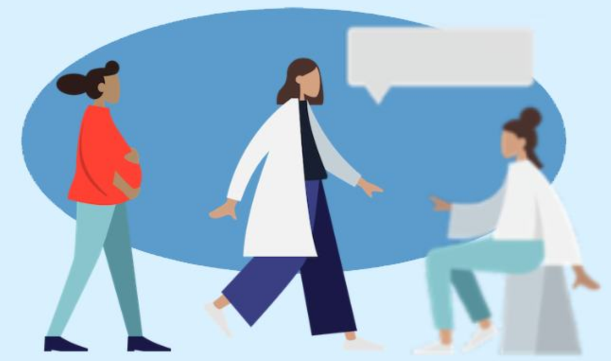

Dr. Smith is American but many of her patients are Latine. She knows that this **cultural divide often makes it difficult to connect with one another and meet each other's care expectations.** Still, Dr. Smith wants to know how she can help close this gap, so she can provide care that's culturally relevant and effective.

DESIGN CHALLENGE

Help Dr. Smith **empathize and connect** with her **Latino/a/e patients' cultural background**

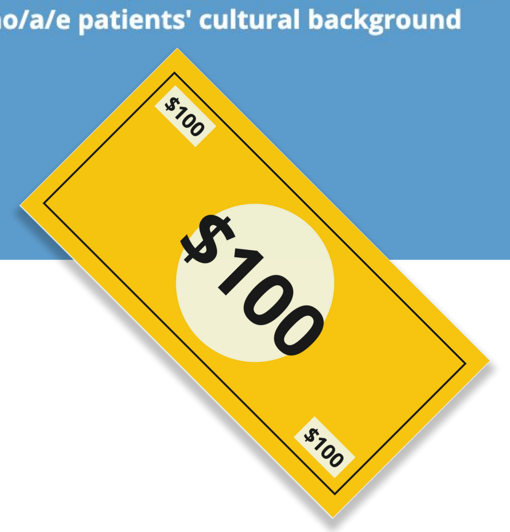

AHL Co-creation | Idea canvas

SESSION

PARTICIPANTS

SKETCH

Draw a sketch, diagram, or make a visual representation of your idea

PURPOSE / VALUE — *See purpose cards*

- *The challenge the idea addresses*
- *How would it help patients or practitioners*

CHARACTERISTICS

- *Words you would use to describe it*
- *How would it make you feel*
- *If the idea was a person it would be...*
- *What it should not be like*

HOW — *See channel cards*

- *How would this idea be delivered? (see "channel" cards)*
- *The activities this idea would involve*
- *Who would be involved in delivering this idea*
- *Any system dependencies for realizing this idea?*

AHL Co-creation | website or digital tool

SESSION

PARTICIPANT

www.

AHL Co-creation | phone app, social media, chat line

SESSION

PARTICIPANT

AHL Co-creation | support person

SESSION

PARTICIPANT

PERSON

- *what are they like?*
- *how do they present themselves?*

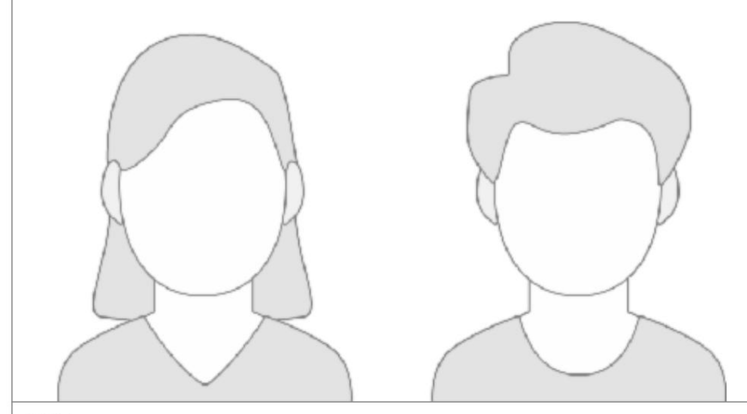

BACKGROUND

- *What cultural influences do they have?*
- *What languages do they speak?*
- *What are they really good at?*
- *What's their passion?*

ROLE

- *How will they help solve the challenge?*
- *What skills do they bring to the table?*
- *Who are they affiliated with?*
- *What would they do on a day to day?*
- *How are they trained?*

PERSONALITY

- *Who would you like them to be like?*
- *What words would you use to describe them to another person?*
