# Supplementary material for: Using human-centered design to advance health literacy in local health department programming: a case study
Source: BMC Public Health. 2025 Mar 31;25:1207. doi: 10.1186/s12889-025-22491-z (PMC11956235; doi:10.1186/s12889-025-22491-z)
Supplement: Supplementary file 4 — Supplementary Material 4 [file 12889_2025_22491_MOESM4_ESM.docx]

### Important Links

- [Community Pilot Plan](https://docs.google.com/document/d/1x2hJUyVX5FESPXtsjXsofIvhNJKdh-nSHYQsiw8axQo/edit)
- [AHL Community Pilot Process Evaluation Plan](https://docs.google.com/document/d/1H1i2KDDE3rdvfmUxmA5HnbdeqZJTem15j7Swv_2GSgE/edit#heading=h.xqtggn3ptf4p)
- [Miro Board of Pilot Workflow](https://miro.com/app/board/uXjVPj7NFGI=/)

### Evaluation Objectives

The workshop objective is for participants to leave knowing appropriate places they can go for different kinds of health care in Pima County. The goals of this survey are to:

- Measure changes in confidence and competence (self-efficacy) to find and use health information including navigating the healthcare system.
- Measure changes in behavioral intent to engage in health seeking behaviors including navigating the healthcare system.
- Explore satisfaction and experience with the interventions including the workshop and WhatsApp groups.

### Pre-Survey Workflow

- Participants use the QR code or web link provided on community fliers to complete the Google registration form. That form asks key demographic questions to determine if they are eligible for our sample and therefore to receive the survey.
- Community partners will create a unique ID for each registered participant (including those not in our sample). The Eval Team will support partners in this process.
- ~1 week before the workshop, promotoras will contact the participants before the workshop and explain the incentive and participation requirements.
- A modified consent form will be included in the first page of the survey. [A paper consent form will be used later in the process, before interviews].
- Promotoras will send registered participants who are in our priority population and eligible to complete the survey via email or SMS link to complete in Qualtrics.

### Post-Survey Workflow

- ~4 weeks after the workshop, the post-survey will be sent via email or SMS link. Qualtrics can link pre-post surveys; the post-survey will be a unique link that is tied to the pre-survey.
- PCHD will monitor pre-post survey completion and notify the Promotoras of who needs reminders.
- Promotoras will send a reminder if they haven’t completed it after 1-2 weeks.
- PCHD will distribute the incentive to the participant.

### Pre-Survey

**Consent**

**Page 1: First/last initial & birthdate**

**Page 2: Would you like to complete this in English or Spanish?**

[Answer options: English, Spanish]

**Page 3: How would you like to receive your compensation?**

[Answer options: I would like a digital gift card emailed to me, I would like a physical gift card by certified mail (which will require a signature for delivery) [open text field for their address], I would like to pick up a physical gift card at Abrams Public Health Center 3950 S. Country Club Rd, Tucson, AZ 85714]

**Page 4. You will now be asked a series of questions about your experience with healthcare. There are no right or wrong answers and you can skip questions you do not want to answer. Please answer to the best of your ability. If you have any questions, ask the person who gave you the survey. This should take you X minutes to complete.**

**Page 5. Below we’ve listed some things people might do for their health. We want to understand if our workshop can help people learn new ways to take care of their health. For each one, let us know if this is something you have done before. If you haven’t, please indicate if you feel comfortable doing it or not.**

Answer options: [I can do this already ✔

This is new and I am comfortable trying this 🙂 or 👍

This is new and I am not comfortable trying this 😟 or 👎

This does not apply to me. ❌]

1. Find useful information and resources for my health
2. Access community resources like health events, classes, and support groups
3. Book or schedule a healthcare appointment
4. Find a COVID-19 vaccine or booster
5. Complete medical forms
6. Discuss my health needs with my medical team
7. Ask for an interpreter, if needed
8. Ask for a doctor or nurse who shares an identity with me (e.g., race, ethnicity, gender)
9. Understand when and how to start or stop taking a medicine
10. Talk to a trusted family member, friend, or community member about my health

**Page 6. What are some of the things that prevent you from taking care of your health and accessing care when you need it?**

1. Please select all that apply:
2. I don’t know where to start.
3. I don’t have a friend or family member who can help me.
4. I am scared or anxious about going to the doctor.
5. I feel like the doctor won’t help me.
6. I have difficulty making an appointment.
7. I’m confused about what is covered by insurance.
8. I can’t afford the cost.
9. I don’t have time.
10. I don’t have transportation.
11. I don’t speak or understand English well.
12. My immigration status is a barrier.
13. Nothing, I feel like I take care of my health well.
14. Other (open text)

**Page 7. Please select the answer that best represents your response.**

[Answer options: Always, Often, Sometimes, Occasionally, Never]

1. How often do you have someone help you read materials related to your health?
2. How often do you have problems learning about your medical condition because you struggle to understand written information?
3. How often do you have a problem understanding what doctors or nurses tell you?
4. How confident are you in filling out medical forms by yourself?

**Page 8. Now we are going to ask some questions about your experiences with your medical team in Tucson. Think about the last few times you have seen a doctor or nurse in Tucson. How often do they:**

[Answer options: Always, Usually, Sometimes, Never, This doesn’t apply to me]

1. Listen carefully to you
2. Explain things so you can understand them
3. Show respect for what you have to say
4. Spend enough time with you
5. Ask you to describe how you will follow the instructions in your own words
6. Offer translation services
7. Confirm the next steps after the visit
8. Follow up after the visit
9. Involve you in decisions about your care

**Page 9. We are interested in learning more about how the COVID-19 pandemic has influenced the lives of Pima County community members. Please select the answer that best represents your response.**

1. Have you received at least one dose or shot of a COVID-19 vaccine? Y/N
2. How many booster shots have you received? 0, 1, 2, 3
3. Has a doctor or nurse, or other health professional ever recommended that you get a COVID-19 vaccine? [Answer options: Yes/No]
4. In the last month, have you seen or heard any negative information about the safety or effectiveness of COVID-19 vaccines? [Answer options: Yes/No]
5. In the last month, have you wanted to learn more information about Covid-19? [Answer options: Yes/No]

*LOGIC [If yes to question 29]*

1. Where did you look for more information? [Answer options: Online search, social media, newspaper, television or radio, asked my my doctor or nurse, asked a family member or friend, other (open text)]
2. Was it easy to find the information you were looking for? [Answer options: Yes/No]
3. Did you take any sort of action afterwards? [Answer options (select all that apply): I shared it with a friend or family member, I shared it on social media, I made an appointment to get vaccinated, I got vaccinated, I made an appointment with my doctor, I did not take any action, other (open text).

**Page 10: Thank you for your responses!**

###

### Post-Survey

**Page 1: First/last initial & birthdate**

**Page 2: Would you like to complete this in English or Spanish?**

[Answer options: English, Spanish]

**Page 3. You will now be asked a series of questions about your experience with healthcare. There are no right or wrong answers and you can skip questions you do not want to answer. Please answer to the best of your ability. If you have any questions, ask the person who gave you the survey. This should take you X minutes to complete.**

**Page 4. Below we’ve listed some things people might do for their health. We want to understand if our workshop helped people learn new ways to take care of their health. For each one, let us know if this is something you have done since the workshop. If you haven’t, please indicate if you feel comfortable doing it or not.**

Answer options: [I can do this already ✔

This is new and I am comfortable trying this 🙂 or 👍

This is new and I am not comfortable trying this 😟 or 👎

This does not apply to me. ❌]

1. Find useful information and resources for my health
2. Access community resources like health events, classes, and support groups
3. Book or schedule a healthcare appointment
4. Find a COVID-19 vaccine or booster
5. Complete medical forms
6. Discuss my health needs with my medical team
7. Ask for an interpreter, if needed
8. Ask for a doctor or nurse who shares an identity with me (e.g., race, ethnicity, gender)
9. Understand when and how to start or stop taking a medicine
10. Talk to a trusted family member, friend, or community member about my health

**Page 5. What are some of the things that prevent you from taking care of your health** and accessing care when you need it?

1. Please select all that apply:
2. I don’t know where to start.
3. I don’t have a friend or family member who can help me.
4. I am scared or anxious about going to the doctor.
5. I feel like the doctor won’t help me.
6. I have difficulty making an appointment.
7. I’m confused about what is covered by insurance.
8. I can’t afford the cost.
9. I don’t have time.
10. I don’t have transportation.
11. I don’t speak or understand English well.
12. My immigration status is a barrier.
13. Nothing, I feel like I take care of my health well.
14. Other (open text)

**Page 6. Please select the answer that best represents your response.** [Answer options: Always, Often, Sometimes, Occasionally, Never]

1. How often do you have someone help you read materials related to your health?
2. How often do you have problems learning about your medical condition because you struggle to understand written information?
3. How often do you have a problem understanding what doctors or nurses tell you?
4. How confident are you in filling out medical forms by yourself?

**Page 7. Now we are going to ask some questions about your experience participating in the "Navigating Healthcare in Pima County" workshop.**

1. **Please provide the date and location of the workshop you attended:**
   1. Time & Date [Drop down or Calendar functionality]
   2. Location [Drop down [insert final [study locations](https://docs.google.com/spreadsheets/d/1ky2P6G546QIyLqeiLGx3ZJSe_qWDBXjx90N0-QYIjHA/edit#gid=0)] and virtually]
2. **For each of the following, let us know how satisfied you were with each.** [Answer options: Very satisfied, Somewhat satisfied, Not at all satisfied, Not applicable]
   1. Signing up for the workshop
   2. Completing the registration form
   3. The content of the workshop
   4. The length of the workshop
   5. The facilitator
   6. The discussion and activities
3. **How useful were the following topics discussed in the workshop?** [Very useful, Somewhat useful, Not at all useful, Not applicable]
   1. Where to go depending on my healthcare needs and situations
   2. How to pay for healthcare
   3. Patient rights (e.g., privacy, asking for a practitioner of a particular sex if you feel comfortable, asking for an interpreter)
4. **Did you join the WhatsApp group?** [Answer options: Yes, No]
5. *LOGIC [If no to question 19]* **Why did you decide not to participate? Select all that apply.**
   1. I felt that I had all the information that I needed after the workshop.
   2. I don’t have the time.
   3. I did not want to provide my phone number.
   4. I was not satisfied with the content of the workshop.
   5. It didn’t seem useful.
   6. I am not comfortable with using WhatsApp.
6. *LOGIC [If yes to question 19]* **How useful has the information provided in the WhatsApp Group been so far?** [Very useful, Somewhat useful, Not at all useful, Not applicable]

**Page 8. We are interested in learning more about how the COVID-19 pandemic has influenced the lives of Pima County community members. Please select the answer that best represents your response.**

1. Have you received at least one dose or shot of a COVID-19 vaccine? Y/N
2. How many booster shots have you received? 0, 1, 2, 3
3. If you haven’t gotten a vaccine or booster, can you tell us why? You can select all that apply and use the text box to share your response. [Answer options: Open text; Select all that apply: I’m already up to date, I’m not eligible for a booster, I’m not required to get one, I don’t want to get vaccinated, My family or friends wouldn’t be happy if I got vaccinated, I couldn’t get the time off work, I couldn’t find childcare, I couldn’t make an appointment, I couldn’t find a vaccine site, I wasn’t sure if it would be free or covered by health insurance, I’m afraid of needles, I’m afraid of the side effects, I already had Covid, I’m not worried about getting it]

**Page 9: Thank you for your responses**
