# Supplementary material for: Using human-centered design to advance health literacy in local health department programming: a case study
Source: BMC Public Health. 2025 Mar 31;25:1207. doi: 10.1186/s12889-025-22491-z (PMC11956235; doi:10.1186/s12889-025-22491-z)
Supplement: Supplementary file 5 — Supplementary Material 5 [file 12889_2025_22491_MOESM5_ESM.docx]

## **Clinical Pilot: Client Pre-Post Surveys**

### Evaluation Objectives

The AHL clinical pilot provides opportunities for clients to engage with three interventions: Health Priorities Guide, Covid Conversations, and the After Visit Summary. The goals of this pre-post survey are to:

- Measure changes in **confidence and competence (self-efficacy)** to find and use health information including navigating the healthcare system, learning about COVID-19, and getting the COVID-19 vaccination.
- Measure changes in **behavioral intent** to engage in health seeking behaviors including navigating the healthcare system and getting the COVID-19 vaccination.
- Measure **satisfaction** with their medical care and the interventions they received.

### Pre-Survey Workflow

- Client interest is gauged in the initial recruitment call. A unique ID is made and recorded in the roster.
- Clients come in for their appointment and a promotora or front desk staff hands them a tablet to complete the consent form, survey and video (if no Promotora is available).
- The promotora or front desk staff will pull up the Survey Monkey link and enter their unique ID on the first page.
- If the promotora is available, they will help the patient walk through the consent form and let the patient know they don’t have to watch the video at the end. MHC will have physical consent forms (and surveys) available for participants to take if they wish, or if the tablets don't work.
- If they are NOT available, the front desk will provide guidance and ensure they watch the video.

### Post-Survey Workflow

- The promotora completes the follow-up call, mentions that a survey will be sent to them soon, and reminds them that they will receive their compensation after they complete it.
- The survey is sent to participants via email or text message.
- MHC staff will send a reminder if they haven’t completed it after one week.
- The completed survey will be recorded in the participant roster.
- MHC will note to PCHD who completed the survey.
- PCHD will distribute the incentive to the participant.

### Pre-Survey

**Page 1: Unique ID [Open text; to be completed by front desk staff or promotora]**

***pages after this would be client facing***

**Page 2: Would you like to complete this in English or Spanish?**

[Answer options: English, Spanish]

**Page 3: English or Spanish Consent Form**

**Page 4: Consent Signature**

**Page 5: How would you like to receive your compensation?**

[Answer options: I would like a digital gift card emailed to me, I would like a physical gift card by certified mail (which will require a signature for delivery) [needs an open text field for their address], I would like to pick up a physical gift card at Abrams Public Health Center 3950 S. Country Club Rd, Tucson, AZ 85714]

**Page 6. You will now be asked a series of questions about your experience with healthcare. There are no right or wrong answers and you can skip questions you do not want to answer. Please answer to the best of your ability. If you have any questions, ask the person who walked you through the consent process. This should take you X minutes to complete.**

**Page 7. Below we’ve listed some things people might do for their health. For each one, let us know if this is something you already do. If you don’t, let us know how confident you would be to do this.**

1. Look up information and resources for my health
2. Look up information and resources for COVID-19 vaccines and boosters
3. Access community resources and tools like health events, classes, and support groups
4. Access community resources and tools for COVID-10 vaccines and boosters like free mobile clinics
5. Book a healthcare appointment with my current provider
6. Book a healthcare appointment with a new provider
7. Book a lab appointment for something like a blood draw or special test
8. Book a COVID-19 vaccine appointment
9. Find a COVID-19 vaccine site
10. Complete medical forms
11. Discuss my medical needs with my healthcare team
12. Ask my healthcare team questions
13. Ask my healthcare team for additional resources and tools
14. Review my next steps and actions items after an appointment
15. Discuss COVID-19 vaccines with my healthcare team
16. Fill a prescription
17. Start or stop a medication
18. Follow the instructions on the label of a medication bottle
19. Talk to a trusted family member, friend, or community member about my health

**Page 8. For the items above that you said “Not at all confident”, could you tell us why? For example, maybe you aren’t sure where to start or would want help from a peer or expert? Or maybe this just isn’t something that your friends or family do often? Does it feel like an overwhelming thing to start? Or maybe it would be difficult to find the time off work or childcare?**

*Alt options for a select all that apply:*

- *Feeling overwhelmed or confused about where to start*
- *Not knowing where to look for resources*
- *Not having a peer or family member to ask for help*
- *Cost*
- *Lack of time*
- *Difficulty getting time off work*
- *Difficulty finding childcare*
- *Fear or anxiety about going to the doctor*
- *Feeling like my doctor won’t listen or care*
- *Not knowing what questions to ask the doctor*
- *Not having enough time to ask the doctor questions*
- *Fear or anxiety about the Covid-19 vaccine*
- *Not knowing where to get vaccinated against Covid-19*
- *Not knowing where to book an appointment or fill a prescription*
- *Confusion over what is covered by my insurance*
- *Anxiety over not being able to cover my portion (co-pay) of the appointment*
- *Anxiety over not being able to cover my portion (co-pay) of prescription (medication)*
- *Difficulty booking an appointment*
- *Transportation issues*
- *Language barriers*
- *Other (open text)*

1. [Answer options: Open text]

**Page 9. Please select the answer that best represents your response.**

[Answer options: Always, Often, Sometimes, Occasionally, Never]

1. How often do you have someone help you read hospital materials?
2. How often do you have problems learning about your medical condition because of difficulty understanding written information?
3. How often do you have a problem understanding what is told to you about your medical condition?
4. How confident are you in filling out medical forms by yourself?

**Page 10. Now we are going to ask several questions about COVID-19 and the COVID-19 vaccine. Please select the answer that best represents your response.**

1. What is your current vaccination status? [Answer options: Up to date on all vaccines and boosters, Need a booster, Unvaccinated, Unsure]
2. In the last 4 weeks, have you wanted to learn more information about Covid-19? [Answer options: Yes/No]

*LOGIC [If yes to question 26]*

1. Where did you look for more information? [Answer options: Online search, social media, asked my healthcare team, asked a family member or friend, other (open text)]
2. What were you looking for? [Answer options: General information, symptoms, treatment options, vaccine information, where to get a vaccine or a booster, other (open text)]
3. Was it easy to find the information you were looking for? [Answer options: Yes/No]
4. *LOGIC [If no to question 29]* Why not? (open text)
5. Did the information feel trustworthy and reliable? [Answer options: Yes/No]
6. *LOGIC [If no to question 31]* Why not? (open text)
7. Did you take any sort of action afterwards? [Answer options: Shared it with a family member or friend, shared it on social media, made an appointment to get vaccinated, got vaccinated, made an appointment with my healthcare provider, no action, other (open text)]

**Page 11. Now we are going to ask some questions about your last healthcare experience.**

1. Have you had a healthcare appointment in the last 12 months? [Answer options: Yes/No; *LOGIC If no*, skip the remaining questions]
2. Where was it? [Answer options: MHC, other (open text)]
3. When was it? [Answer options: month/year, unsure]
4. What was it for? [Answer options: Annual physical, Annual Ob-Gyn check-up, Specialist visit, Pregnancy care, Vaccination, other (open text)]
5. During that appointment, did your healthcare team: [Answer options: Always, Usually, Sometimes, Never]
   1. Listen carefully to you
   2. Explain things so you could understand them
   3. Show respect for what you had to say
   4. Spend enough time with you
   5. Ask how instructions will be followed
6. Did you feel you were involved in decisions about your health care as much as you wanted? [Answer options: Always, Usually, Sometimes, Never]

**Page 12: Thank you for your responses! Now, it is time to prepare for your appointment. Please watch either the English or Spanish video below and complete the Health Priorities Worksheet before your appointment. If you have any questions, ask a staff member.**

[Spanish Video](https://www.youtube.com/watch?v=8I_cwRkBuH0)

[English Video](https://www.youtube.com/watch?v=tgY-Z32yBTY)

### Post-Survey

**Page 1: Would you like to complete this in English or Spanish?**

[Answer options: English, Spanish]

**Page 2. You will now be asked a series of questions about your experience with healthcare. There are no right or wrong answers and you can skip questions you do not want to answer –– please answer to the best of your ability. If you have any questions, feel free to reach out to (names of the promotoras). This should take you X minutes to complete.**

**Page 3. Below we’ve listed some things people might do for their health. For each one, let us know if you have done this since your last healthcare appointment. If you haven’t, let us know how confident you would be to do this.**

[Answer options (only 1 option allowed): I did this, This isn’t relevant for me, Very confident I could do this, Pretty confident I could do this, Somewhat confident I could do this, Not at all confident I could do this, Not sure]

1. Look up information and resources for my health
2. Look up information and resources for COVID-19 vaccines and boosters
3. Access community resources and tools like health events, classes, and support groups
4. Access community resources and tools for COVID-10 vaccines and boosters like free mobile clinics
5. Book/schedule a healthcare appointment with my current provider
6. Book/schedule a healthcare appointment with a new provider
7. Book/schedule a lab appointment for something like a blood draw or special test
8. Book/schedule a COVID-19 vaccine appointment
9. Find a COVID-19 vaccine site
10. Complete medical forms
11. Discuss my medical needs with my healthcare team
12. Ask my healthcare team questions
13. Ask my healthcare team for additional resources and tools
14. Review my next steps and actions items after an appointment
15. Discuss COVID-19 vaccines with my healthcare team
16. Fill a prescription
17. Start or stop a medication
18. Follow the instructions on the label of a medication bottle
19. Talk to a trusted family member, friend, or community member about my health

**Page 4. For the items above that you said “Not at all confident”, could you tell us why? For example, maybe you aren’t sure where to start or would want help from a peer or expert? Or maybe this isn’t something that your friends or family do often? Does it feel like an overwhelming thing to start? Or maybe it would be difficult to find the time off work or childcare?**

1. [Answer options: Open text]

**Page 5. Please select the answer that best represents your response.**

[Answer options: Always, Often, Sometimes, Occasionally, Never]

1. How often do you have someone help you read hospital materials?
2. How often do you have problems learning about your medical condition because of difficulty understanding written information?
3. How often do you have a problem understanding what is told to you about your medical condition?
4. How confident are you in filling out medical forms by yourself?

**Page 6. Now we are going to ask some questions about your last healthcare appointment at MHC.**

1. Do you remember receiving any of the following? [Answer options: My Health Priorities guide, Visit Summary Checklist]
2. Do you remember talking with your healthcare team about the COVID-19 vaccine? [Answer options: Yes/No]
3. During that appointment, did your healthcare team: [Answer options: Always, Usually, Sometimes, Never]
   1. Listen carefully to you
   2. Explain things so you could understand them
   3. Show respect for what you had to say
   4. Spend enough time with you
   5. Ask how instructions will be followed
4. Did you feel you were involved in decisions about your health care as much as you wanted? [Answer options: Always, Usually, Sometimes, Never]
5. For each of the following, let us know how satisfied you were with each. [Answer options: Very satisfied, Pretty satisfied, Somewhat satisfied, Not at all satisfied, Not applicable]
   1. Booking the appointment
   2. Completing medical paperwork and forms
   3. Watching the Promotora video in the wait room
   4. Appointment length
   5. My Health Priorities guide
   6. Ability to ask questions to your health team
   7. Visit Summary Checklist
   8. Follow-up call with the CHW/promotora
   9. Making a follow-up appointment
   10. Other (open text)

**Page 7. Now we are going to ask several questions about COVID-19 and the COVID-19 vaccine. Please select the answer that best represents your response.**

1. What is your current vaccination status? [Answer options: Up to date on all vaccines and boosters, Need a booster, Unvaccinated, Unsure]
2. Since your last appointment, have you done any of the following? [Answer options: Got vaccinated, Got a booster, Scheduled an appointment to get the vaccine or booster, Discussed the vaccine or booster with my healthcare team, Discussed the vaccine or booster with a friend or family member, Looked for more information, Attended an event about the vaccine or booster, following up with a resource provided by the community health worker/provider, I haven’t done anything]
3. If you didn’t get the vaccine or a booster, can you tell us why? You can select all that apply and use the text box to share your response. [Answer options: Open text; Select all that apply: I’m already up to date, I don’t want to get vaccinated, My family or friends wouldn’t be happy if I got vaccinated, I couldn’t get the time off work, I couldn’t find childcare, I couldn’t make an appointment, I couldn’t find a vaccine site, I wasn’t sure if it would be free or covered by health insurance, I’m afraid of needles, I’m afraid of the side effects]

**Page 12: Thank you for your responses!**
