# Supplementary material for: Using human-centered design to advance health literacy in local health department programming: a case study
Source: BMC Public Health. 2025 Mar 31;25:1207. doi: 10.1186/s12889-025-22491-z (PMC11956235; doi:10.1186/s12889-025-22491-z)
Supplement: Supplementary file 6 — Supplementary Material 6 [file 12889_2025_22491_MOESM6_ESM.docx]

**AHL: Community Pilot – Guidelines and Interview Questions**

**Workshop Participant**

**Welcome and Ground Rules**

Good morning/evening, and welcome. Thanks for taking the time to join me/us for the discussion about the Navigating Healthcare Workshop. My name is XXXXX. We will first begin by going over the consent form. (Take 5 – 10 minutes reviewing the consent form with participant and answering any questions. Remember, both you and the participant need to sign two forms, you will keep one and the participant will keep the other.)

Now, as we begin with the interview, remember, there are no wrong answers. We expect that individuals participating in these interviews will have differing points of view. Please share your point of view. This interview will take about 1 hour. If there are questions you prefer not to answer, please let me know, and we can move on to the next question.

I am recording the session because I don’t want to miss any of your comments. No names will be included in any reports. Your comments are confidential.

I am here to ask questions and listen.

If you have a cell phone, please put it on quiet mode, and if you need to answer, step out to do so. Let’s get started with the first question.

**Workshop Participant Questions**

1. Did you complete the workshop in person or virtually? Who was your facilitator?
2. Please share your experience in participating in the Navigating Healthcare Workshop. What are some of the things you learned from the workshop?
3. After attending the Navigating Healthcare Workshop, please describe any changes to your confidence in accessing healthcare.
   1. Possible probe: Did you experience any challenges with making these changes? (e.g., time constraints, awkward interactions, frustration with the interaction, etc.)
   2. Possible probe: Did you notice any changes in your strategies/approaches to accessing healthcare? (e.g., more targeted conversation, working with your doctor to find the best solution to your concern, feeling heard and understood, less stress or tension when reaching out to healthcare providers or asking about a different approach to your concern, etc.)
4. Did you observe any changes in your interaction with the provider(s) once you accessed healthcare? (e.g., you were able to ask more questions, changes in your provider’s mannerisms, the provider was more responsive to involving you in decisions about your health, etc.)
5. If you opted to participate in the WhatsApp group chat, please share your experience with us. If you didn’t opt into the WhatsApp group chat, please tell us about your decision not to participate.
6. Since attending the Navigating Healthcare Workshop, how often have you visited the doctor? How have these experiences changed or stayed the same since taking the Navigating Healthcare Workshop?
7. After attending the Navigating Healthcare Workshop, how confident do you feel in paying or figuring out how to pay for healthcare?
   1. What are some of the tools that you learned about and used to pay for healthcare that was talked about in the workshop?
   2. From what you have learned in the workshop, what are some ways for paying for healthcare that you have utilized?
8. Anything else you would like to add that might help us as we move forward in this study?

**Facilitator Interview Questions**

**Community Pilot**

**Navigating Healthcare Workshop**

Name of Facilitator:

Date:

Name of Interviewer:

1. Tell me about your position at PPEP/LC?
2. Did you facilitate the workshops online or in person?
3. What was your experience in facilitating online/in person?
4. Can you describe the primary goals of the Navigating Healthcare workshop and how they align with empowering participants to make informed healthcare choices?
5. How do you ensure that participants leave the workshop with a clear understanding of appropriate places they can go for healthcare and their eligibility for different healthcare options?
6. How do you measure the effectiveness of the workshop in terms of behavior change? Can you provide examples of how participants have applied what they learned to find and use health information or access new healthcare resources?
7. Please describe your experience with using WhatsApp groups/communities during the pilot.
   1. Did you notice any changes in the participant’s viewpoints or experiences accessing healthcare as you interacted in the WhatsApp groups/communities?
   2. What were some challenges in using WhatsApp?
   3. What were some successes that you witnessed while interacting with participants in the WhatsApp groups/communities?
8. Can you share any success stories or anecdotes from previous workshop participants who were able to find and use health information or access healthcare options they didn't have access to before attending the workshop?
9. How do you adapt the workshop content and delivery to the specific needs of your audience, considering factors like demographics, cultural background, and literacy levels?
10. What challenges have you encountered in facilitating the Navigating Healthcare workshop, and how have you addressed them to ensure that participants leave with the knowledge and abilities they need?
11. Do you think that your organization would be able and/or willing to continue having the Navigating Healthcare Workshops without the support of the Pima County Health Department?
12. What was your experience working with PCHD as partners? Did you feel supported? What are some ways that you think PCHD can improve their partnership with your organization?
13. Is there anything else that I might have missed that you would like to share with right now?
