# Supplementary material for: Using human-centered design to advance health literacy in local health department programming: a case study
Source: BMC Public Health. 2025 Mar 31;25:1207. doi: 10.1186/s12889-025-22491-z (PMC11956235; doi:10.1186/s12889-025-22491-z)
Supplement: Supplementary file 7 — Supplementary Material 7 [file 12889_2025_22491_MOESM7_ESM.docx]

**AHL: MHC Client Post-Intervention Guidelines and Interview Questions**

**Welcome and ground rules**

Good morning/evening and welcome. Thanks for taking the time to join me/us for the discussion about the AHL program. My name is XXXXX.

There are no wrong answers. We expect that individuals participating in these interviews will have differing points of view. Please share your point of view. This interview will take about 1 hour. If there are questions you prefer not to answer, please let me know and we can move on to the next question.

I am recording the session because I don’t want to miss any of your comments. No names will be included in any reports. Your comments are confidential.

I am here to ask questions and listen.

If you have a cell phone, please put it on the quiet mode, and if you need to answer, step out to do so. Let’s get started with the first question.

**MHC Client Questions**

1. Thinking back to your interactions with your healthcare providers, how would you describe your routine engagement with your provider(s)? Has your routine engagement with your provider(s) changed in the past year?
2. After attending the enhanced intervention at MHC, please describe any changes to your interactions with your provider(s).
   1. Possible probe: Did you experience any challenges with making these changes? (e.g., time constraints, awkward interactions, frustration with the interaction, etc.)
   2. Possible probe: Did you notice any changes in your strategies/approaches with making these changes? (e.g., more targeted conversation, working together to find the best solution to your concern, feeling heard and understood, less stress or tension when asking follow-up questions or asking about a different approach to your concern, etc.)
   3. Possible probe: Did you observe any changes in your interaction with the provider(s)? (e.g., a more engaged provider, more questions being asked, changes in your provider’s mannerisms, the provider was more responsive to involving you in decisions about your health, etc)
3. Could you describe any examples of your provider taking your cultural viewpoint into consideration when engaging with you during your appointment?
4. Will you continue to use the goal tool for future appointments? What were some of the things you learned or interactions with the community health worker or other MHC providers that you thought were useful?
   1. Possible probes: were you satisfied with the enhanced intervention you received? How acceptable and useful are these tools and communication techniques?
5. If you could make changes or recommendations on the enhanced intervention, what would they be?
   1. Possible probes: Add tools, take away a tool or section, add different communication prompts on the provider side, more time with the community health worker, more check-in appointments with the community health worker, etc.
6. Is there anything else you would like to share with us that has not already been covered?

**Closing Statement**

Thank you, that concludes our interview. We value your perspective and recognize how busy you are and we are very appreciative that you took the time to spend this hour speaking with us. If any questions come up and you would like to follow up with me please feel free to call me at (xxx) xxx-xxxx or you can email me at [xxx@arizona.edu](mailto:xxx@arizona.edu).

**AHL: Provider Post-Intervention Interview Questions**

**Welcome and ground rules**

Good morning/evening and welcome. Thanks for taking the time to join me/us for the discussion about the AHL program. My name is XXXXX.

There are no wrong answers. We expect that individuals participating in these interviews will have differing points of view. Please share your point of view. This interview will take about 1 hour. If there are questions you prefer not to answer, please let me know and we can move on to the next question.

I am recording the session because I don’t want to miss any of your comments. No names will be included in any reports. Your comments are confidential.

I am here to ask questions and listen.

If you have a cell phone, please put it on the quiet mode, and if you need to answer, step out to do so. Let’s get started with the first question.

**Provider Questions**

1. Thinking back to your day-to-day patient interactions before you attended the CLAS trainings, how would you describe your routine engagement with your patients? Has your routine engagement with your patients changed in the past year?
2. After attending the CLAS trainings, please describe any changes to your interactions with your patients.
   1. Possible probe: Did you experience any challenges with making these changes? (e.g., time restraints, awkward interactions, shifts in your workflow, etc.)
   2. Possible probe: Did you observe any changes in your interaction with the patient? (e.g., a more engaged patient, more questions being asked, changes in your or the patient’s mannerisms, patient is more involved in decisions about their health, etc)
3. Were there steps you took to assess the patient’s cultural viewpoint?
   1. Possible probe: Did you spend time reflecting on your own cultural viewpoint during or after your CLAS training?
   2. Possible probe: If yes to the previous question, how did this self-reflection play a role in how you engaged with patients?
4. Will you continue to use the communication techniques you learned? What steps have you taken to integrate the communication techniques learned through the CLAS trainings?
   1. Possible probes: were you satisfied with attending CLAS trainings? How acceptable, feasible and sustainable are these communication techniques?
5. If you could make changes or recommendations on integrating these communication techniques within your everyday workflow, what would they be?
6. Were there any aspects you learned during the CLAS trainings that would be challenging to integrate into your everyday workflow? If so, why?

**Additional Questions**

1. Thoughts on having a promotoras in the clinics and if you think they will be received well?
2. Any additional comments that you would like to add?
3. What do you think are some steps from the pilot that you would like to keep? What are some steps that you would like to remove?
4. Realistically, how likely do you think MHC would benefit from, or not benefit from, keeping the pilot?

**Closing Statement**

Thank you, that concludes our interview. We value your perspective and recognize how busy you are and we are very appreciative that you took the time to spend this hour speaking with us. If any questions come up and you would like to follow up with me please feel free to call me at (xxx) xxx-xxxx or you can email me at [xxx@arizona.edu](mailto:xxx@arizona.edu).
